# Courage in Decision Making: A Mixed-Methods Study of COVID-19 Vaccine Uptake in Women of Reproductive Age in the U.K.

**DOI:** 10.3390/vaccines12040440

**Published:** 2024-04-18

**Authors:** Laura A. Magee, Julia R. Brown, Vicky Bowyer, Gillian Horgan, Harriet Boulding, Asma Khalil, Nathan J. Cheetham, Nicholas R. Harvey, COVID Symptom Study Biobank Consortium, RESILIENT Study Group, Hiten D. Mistry, Carole Sudre, Sergio A. Silverio, Peter von Dadelszen, Emma L. Duncan

**Affiliations:** 1 School of Life Course & Population Science, King’s College London, London WC2R 2LS, UK; gillian.horgan@kcl.ac.uk (G.H.); hiten.mistry@kcl.ac.uk (H.D.M.); sergio.silverio@kcl.ac.uk (S.A.S.); pvd@kcl.ac.uk (P.v.D.); 2 Department of Twin Research and Genetic Epidemiology, King’s College London, London SE1 7EH, UK; julia.3.brown@kcl.ac.uk (J.R.B.); vicky.bowyer@kcl.ac.uk (V.B.); nathan.cheetham@kcl.ac.uk (N.J.C.); emma.duncan@kcl.ac.uk (E.L.D.); 3 The Policy Institute, King’s College London, London WC2B 6LE, UK; harriet.boulding@kcl.ac.uk; 4 Department of Obstetrics and Maternal Fetal Medicine, St. George’s University of London, London SW17 0RE, UK; asmakhalil79@googlemail.com; 5 Centre for Medical Image Computer, Department of Computer Science, University College London, London WC1E 6BT, UK; c.sudre@ucl.ac.uk; 6 School of Psychology, Liverpool John Moores University, Liverpool L3 5AH, UK

**Keywords:** vaccination, hesitancy, COVID-19

## Abstract

COVID-19 vaccination rates are lower in women of reproductive age (WRA), including pregnant/postpartum women, despite their poorer COVID-19-related outcomes. We evaluated the vaccination experiences of 3568 U.K. WRA, including 1983 women (55.6%) experiencing a pandemic pregnancy, recruited through the ZOE COVID Symptom Study app. Two staggered online questionnaires (Oct–Dec 2021: 3453 responders; Aug–Sept 2022: 2129 responders) assessed reproductive status, COVID-19 status, vaccination, and attitudes for/against vaccination. Descriptive analyses included vaccination type(s), timing relative to age-based eligibility and reproductive status, vaccination delay (first vaccination >28 days from eligibility), and rationale, with content analysis of free-text comments. Most responders (3392/3453, 98.2%) were vaccinated by Dec 2021, motivated by altruism, vaccination supportiveness in general, low risk, and COVID-19 concerns. Few declined vaccination (by Sept/2022: 20/2129, 1.0%), citing risks (pregnancy-specific and longer-term), pre-existing immunity, and personal/philosophical reasons. Few women delayed vaccination, although pregnant/postpartum women (vs. other WRA) received vaccination later (median 3 vs. 0 days after eligibility, *p* < 0.0001). Despite high uptake, concerns included adverse effects, misinformation (including from healthcare providers), ever-changing government advice, and complex decision making. In summary, most women in this large WRA cohort were promptly vaccinated, including pregnant/post-partum women. Altruism and community benefit superseded personal benefit as reasons for vaccination. Nevertheless, responders experienced angst and received vaccine-related misinformation and discouragement. These findings should inform vaccination strategies in WRA.

## 1. Introduction

The first authorised vaccine against SARS-CoV-2 was administered to the U.K. general public on 10 December 2020, initiating the U.K. COVID-19 vaccination campaign. Delivery was prioritised for individuals at high risk of exposure (e.g., healthcare workers) or of serious COVID-19-related complications (e.g., immune-suppressed individuals). Vaccination was subsequently delivered on an age-tiered basis. Eligibility for women of reproductive age (WRA) began on 13 April 2021 for women aged 40–49 years, 13 May 2021 for women aged 30–39 years, and 8 June 2021 for women aged 18–29 [1]. 

In the U.K., pregnant women were not originally included as clinically vulnerable [2] despite recognition of their higher risk for severe COVID-19, increased maternal mortality, and higher pregnancy-related complications (e.g., pre-eclampsia, preterm birth, and perinatal mortality) [3,4,5,6]. On 24 March 2021, pregnancy-specific COVID-19 vaccination information was issued by The Royal College of Obstetricians and Gynaecologists (RCOG), the Royal College of Midwives (RCM), the U.K. Teratology Information Service (UKTIS), and the MacDonald Obstetric Medicine Society (MOMS) [7]. Pregnant women were advised that they could be vaccinated or wait for more information. On 16 April 2021, advice specifically mentioning pregnancy was first issued by the U.K. Joint Committee on Vaccination and Immunisation (JCVI), which did not recognise pregnant women as vulnerable. It was not until 20 August 2021 that the RCOG, RCM, and UKTIS recommended pregnant women be included as “vulnerable”. 

Uptake of at least one dose of COVID-19 vaccine by pregnant women in England increased from <5% in May 2021 to 41.3% by October 2021 [8]. However, vaccine uptake in pregnant women was lower than uptake in WRA considered overall (≈75% in October 2021 [9]). Importantly, ≈25% of critically ill COVID-19 patients were unvaccinated pregnant women at this time [10]. Thus, on 16 December 2021, the JCVI announced that pregnant women were considered “vulnerable” and advised, “Vaccination in pregnancy is strongly recommended”. This recommendation continues and includes ongoing booster doses [11].

Accumulating data support the safety of vaccination during pregnancy with regard to maternal adverse effects and pregnancy outcomes [12]. Nonetheless, any COVID-19 vaccination by the time of birth has plateaued at ≈75%, and of the 25% unvaccinated by birth, fewer than <1% are subsequently vaccinated postpartum [13]. 

Here, we present data from a cohort of WRA, including women considering pregnancy or currently pregnant/postpartum. We evaluate vaccination timing relative to age-based availability and reproductive status, as well as reasons for/against vaccination, aiming to inform general vaccination strategies in WRA.

## 2. Materials and Methods

Recruitment of participants was from the King’s College London-COVID Symptom Study (KCL-CSS)/ZOE app (Research Ethics Committee [REC] reference LRS-19/20-18210) and the COVID Symptom Study Bank (CSSB, REC reference 20/YH/0298). The KCL-CSS/ZOE app was launched jointly by ZOE Ltd. (London, UK) and KCL researchers on 24 March 2020 to track SARS-CoV-2 testing, symptoms, or care [14]. The COVID Symptom Study Biobank (CSSB) was established in September 2020 to support research into the impact and effects of COVID-19, particularly long-illness duration. (For methodological detail, see Supplementary Materials-Methods).

### 2.1. “Pregnancy Planning, the Pandemic, and Me” Study

This study was approved by the CSSB governance committee in May 2021 (ref 0043).

Potentially eligible participants were CSSB WRA (age 18–50 years). Additionally, all WRA who were current ZOE app users were invited by personal e-mail to join the CSSB for this study. The invitation highlighted the aim to capture views of WRA (particularly women considering pregnancy, currently pregnant, or recently pregnant) regarding the pandemic and COVID-19 vaccination. Trial invitations were initially delivered to 1000 women; subsequently, invitations were sent to the remaining eligible ZOE cohort, with one reminder to non-responders two weeks later. Recruitment closed on 1 December 2021.

After the provision of detailed study information and informed consent, responders completed an online questionnaire (Qualtrics platform, Qualtrics, Provo, Utah, USA). All responders who commenced the first questionnaire were invited, by personal e-mail nine months later (August 2022), to the second questionnaire (REDCap web application), timed so that any woman pregnant at the time of the first questionnaire would have given birth.

The first questionnaire assessed demographic data; pregnancy status and health, including mental health; preventative measures against SARS-CoV-2 infection; SARS-CoV-2 testing; previous COVID-19; and COVID-19 vaccination. The second questionnaire requested updated information regarding health status (including any COVID-19), SARS-CoV-2 vaccination status, and pregnancy outcomes and birth experiences. Both questionnaires included open-ended questions with free-text responses. Up to three reminders were sent to non-responders. Data collection closed on 16 September 2022. Both questionnaires are provided in Supplementary Materials-Questionnaires. 

Questionnaire completion was defined a priori as ≥95%, not including qualitative responses. Missing data were not replaced. Ours was a convenience sample of CSSB and ZOE app users.

### 2.2. Analyses

Descriptive analyses included baseline demographics and past history (reproductive and medical), prior SARS-CoV-2 infection, any COVID-19 vaccination (including type and date), and reasons for or against vaccination. Qualitative analysis was performed of invited free-text comments. 

Vaccination timing was considered relative to eligibility (Table S3). Early access to vaccination was offered to frontline healthcare workers (from 14 January 2021), clinically extremely vulnerable individuals (from 18 January 2021), and those who were shielding (15 February 2021). Although we asked whether individuals had health conditions that required shielding, we did not ascertain clinically extreme vulnerable status (noting that government-defined criteria did not include pregnant or postpartum women). The first vaccination was considered delayed if received >28 days from the date of eligibility. We considered the second vaccination to be delayed if received >4 months after the first vaccination, noting that contemporaneous U.K. guidelines recommended a three-month interval between the first and second vaccination.

Free-text data from both questionnaires were analysed together, using directed content analysis [15], whereby coding was structured using pre-determined codes based on quantitative data; codes were sub-categorised as data richness allowed. New codes were assigned for data not covered by pre-existing codes and relevant to COVID-19 vaccination. Findings were ordered in decreasing frequency of occurrence and considered by reproductive (pandemic pregnancy; previous pre-pandemic pregnancies only; never pregnant) status and vaccination delay.

## 3. Results

Of the 85,092 WRA invited to participate, 3568 (4.2%) consented and commenced the first questionnaire (from 7 September to 1 December 2021); 3453/3568 (96.8%) completed the first questionnaire, of whom 2129/3453 (61.7%) completed the second (from 22 August to 16 September 2022), with 35/3453 (1.0%) women completing only the second questionnaire.

### 3.1. Characteristics of Responders

Generally, responders to the first questionnaire were in their late 30s and overweight (noting some women were pregnant) (Table 1). Almost all were white, and few (<10%) were in the most deprived IMD quintile. Approximately 20% were healthcare workers. Past histories included low rates of conditions increasing the risk of severe COVID-19 (e.g., diabetes). Fewer than half had prior COVID-19, with few requiring hospitalisation. Prior mental health problems were commonly reported; however, few women reported current symptoms consistent with a major depressive disorder (i.e., Patient Health Questionnare-2 score ≥3) or generalised anxiety (i.e., Generalised Anxiety Disorder 2-item score ≥3). 

Over two-thirds of the women had been pregnant pre-pandemic. Approximately half had suffered early pregnancy loss; however, most women were parous, with almost all having had a prior livebirth. A minority of women were currently pregnant or postpartum, and a similar proportion were trying to conceive. 

The demographics of the first questionnaire completers were similar to overall ZOE and CSSB invitees (Table S4); other complete responders were more often healthcare workers. 

Responders to the second questionnaire were similar to the first (Table 1), although more had experienced SARS-CoV-2 infection, and more women had experienced at least one pregnancy.

### 3.2. COVID-19 Vaccination

Most questionnaire responders had been vaccinated, with a similar pattern of vaccine type reported by first and second questionnaire responders (Table 2). The Pfizer vaccine was received twice as often as the Astra-Zeneca vaccine. The Moderna vaccine rose in use for boosters (third or subsequent doses).

For women accepting vaccination, the reasons were similar among first and second questionnaire responders (Table 3). Most were worried about getting COVID-19 and related morbidity and, particularly in the first questionnaire, wanted to be active in their communities again. Most (>80%) expressed altruistic reasons for vaccination (e.g., as a community member, to promote herd immunity, and to minimise SARS-CoV-2 spread) and that vaccination benefits outweighed risks, perceived to be minimal. Almost all women who accepted COVID-19 vaccination supported vaccination in general. Planning pregnancy or receiving fertility treatment were not given as reasons for initial or subsequent vaccination.

The sole reason given by most women who had not accepted COVID-19 vaccination by the time of the first questionnaire was concern about long-term side effects (Table 3). None expressed being against all vaccination. Of those responders who remained unvaccinated by the time of the second questionnaire (20/2129, 0.9%), most reported feeling that they were immune and/or having concerns about short- and long-term risks. They also felt that they did not have enough information and/or stated personal belief/philosophical reasons for not accepting the COVID-19 vaccine, although they did not self-identify as being against all vaccination. A small number of women offered planning pregnancy or receipt of fertility treatment as a reason for non-vaccination, but more were concerned that the vaccine might adversely affect pregnancy and/or breastfeeding or had received healthcare provider advice against vaccination (Table 3).

Table 4 (and Figure S1a–d) shows the time to first vaccination for the vaccinated cohort overall (excluding unvaccinated women [n = 44], women who had received early vaccination in trials [n = 14], and women missing vaccination dates [n = 6]). There was little evidence of vaccine delay: over 93% were vaccinated within 28 days of age-based eligibility, at a median of -7 days (i.e., a week earlier than eligibility). 

Excluding women eligible for early access due to occupation or clinical vulnerability, 92.8% of the “general community” were vaccinated within 28 days, at a median of one day after eligibility (Figure S1b). Vaccine delay was not evident among pregnant/postpartum women, if pregnant/postpartum at any time during the pandemic (90.3% vaccinated within 28 days, a median of 1 day after eligibility) (Figure S1c), or if pregnant/postpartum specifically at the time of vaccine eligibility (Figure S1d). Although there were no differences in delay, women who were pregnant/postpartum were slightly more delayed in receiving vaccination (Figure S2a) compared with other women in the general community (Figure S2b) (median time from eligibility of 3 vs. 0 days, *p* < 0.0001). 

There was no evidence of delay for the second vaccination (i.e., everyone who received a first dose subsequently received their second dose within four months.

### 3.3. Directed Content Analysis

Overall, 852 women provided free-text comments to open-ended questions, with 167 women providing 171 comments relating to vaccination (64 comments from the first questionnaire and 107 from the second questionnaire, with four women providing comments in both). Most of these women (n = 145/167, 86.8%) had experienced a pandemic pregnancy (from February 2020 until the second questionnaire administration at the end of August 2022), with fewer having experienced only pre-pandemic pregnancy (n = 14/167, 8.4%), or never pregnant (n = 7/167, 4.2%); one woman’s status was unclear. Most women (n = 142/167, 85.0%) were not vaccine-hesitant. No unvaccinated women provided comments.

Directed content analysis revealed four key themes: (i) Strong Motivation to be Vaccinated; (ii) Adverse Effects of COVID-19 Vaccination; (iii) Misinformation, Misleading Information, and Ever-Changing Information; and (iv) Complicated Decision Making (illustrative quotations, Table 5). 

Most strongly supported was “Strong Motivation to be Vaccinated”, particularly among women with pandemic pregnancy and non-hesitant women. Women highlighted passive immunity transferred through breastfeeding. They noted the challenging logistics of vaccination and, in contrast, the greater ease that would be afforded by vaccination within maternity services (vs. separately delivered). Of note was the self-advocacy, personal research, and persistence required to achieve vaccination, often in the face of inadequate support from clinic and vaccination centre staff; indeed, one woman reported needing the intervention of her Member of Parliament (MP) to access vaccination.

Concerns about “Adverse Effects of COVID-19 Vaccination” related to vaccination itself (e.g., local reactions) and reproductive effects of vaccination for themselves, their pregnancy, and/or their baby. Concerns extended to menstrual irregularities, subfertility, and the possibility that prior pregnancy complications, particularly miscarriage but also others (e.g., twins), may have been related to prior COVID-19 vaccination. Of note, two-thirds of those with pandemic pregnancies who were not vaccine-hesitant still expressed concerns about COVID-19 vaccination.

Women reported receiving “Misinformation, Misleading information, or Ever-changing Information” from government sources and healthcare workers, from vaccine centres to midwifery and primary care staff. Some healthcare providers actively discouraged pregnant women from vaccination or provided advice that the women regarded as poor. Women cited the ever-changing guidance from government and official bodies as further cause for concern. Women also highlighted that pregnancy was an exclusion criterion during early vaccination trials, which conflicted with subsequent government recommendations for vaccination during pregnancy. Consequently, women expressed the need to do their own research and make autonomous decisions. They found this difficult and challenging—our last theme: “Complicated Decision-Making” (Table 5). Of note, the vast majority of women who struggled with their decisions regarding vaccination were not vaccine-hesitant, regardless of pregnancy status.

## 4. Discussion

### 4.1. Summary of Findings

Among U.K. KCL-CSS/ZOE app users in 2021, we heard from approximately 3500 responders to our questionnaire about pregnancy and the pandemic. Almost all had been vaccinated against SARS-CoV-2. There was little evidence of vaccine delay, with even 89% of pregnant women vaccinated promptly once eligible. While women who were pregnant/postpartum were slightly more delayed by the time of actual vaccination (on average, 3 days), the clinical impact of this, personally or at the population level, is unclear. 

The vast majority of participants gave altruistic reasons for vaccination against SARS-CoV-2, were supportive of vaccination in general, perceived benefits as outweighing risks, and were concerned about getting COVID-19. The small number of women declining vaccination most frequently cited concerns about adverse effects, short- and longer-term; about half cited they were already immune. 

Nonetheless, despite being highly motivated towards vaccination (including during pregnancy) and even in those without delay, many women expressed concerns about vaccine safety and the robustness of the information they had received, including from normally respected sources, and found their decision to accept vaccination difficult. Some women drew relationships between vaccination (even pre-pregnancy) and adverse reproductive outcomes (including pregnancy complications); however, others recognised the benefit of vaccination in protecting pregnant women as a high-risk population for severe COVID-19. Worryingly, several had been advised against vaccination by healthcare providers. 

### 4.2. Findings in Relation to the Literature

Relative to other countries, the U.K. was reluctant to recommend COVID-19 vaccination during pregnancy. Following JCVI advice (24 March 2021), the RCOG, RCM, UKTIS, and MOMS issued (jointly) a several-page information sheet for COVID-19 vaccination in pregnancy [7], in which women were offered the following choices: “Get a COVID-19 vaccine” or “Wait for more information about the vaccine in pregnancy”, presented of equal validity, without editorial comment promoting vaccination. The RCOG and RCM recommended COVID-19 vaccination in pregnancy in August 2021 and the U.K. JCVI on 16 December 2021 after it was clear that unvaccinated pregnant women were over-represented among critically ill patients with COVID-19 [16]. 

In contrast, other international societies recommended COVID-19 vaccination earlier. For example, on 18 December 2020, the Society of Obstetricians and Gynaecologists of Canada recommended offering COVID-19 vaccination, citing, “…the risk of infection and/or morbidity from COVID-19 outweighs the theorized and undescribed risk of being vaccinated during pregnancy or while breastfeeding”, and cited decades of experience with other vaccines administered in pregnancy [17], while also leaving open the possibility of revised advice based on emerging evidence. In contrast, responders to our questionnaire described their (and healthcare providers’) struggles with U.K. advice, which either left *them* with the obligation to decide and/or changed without clear explanation. In the future, it may be useful to revisit the obligation of the RCOG and RCM to follow JCVI’s direction regarding vaccination recommendations. 

Our responders’ concerns about prior vaccination and pregnancy complications highlight the need to provide and promote greater health literacy about reproductive outcomes, including baseline risks. For example, miscarriage complicates about 10% of recognised conceptions [18]; if the vast majority of the population is being actively vaccinated, then vaccination and miscarriage will occur near-contemporaneously commonly, by chance alone. Social media promoted the concept that vaccination caused miscarriage, with messages featuring potential cross-reactivity of SARS-CoV-2 spike protein antibodies (following vaccination with mRNA COVID-19 vaccines) with syncytin-I protein in trophoblast cells raising concerns that COVID-19 vaccination could harm placental tissue despite low homology between spike proteins and syncytin-I and the lack of supporting epidemiological data [19]. Despite reassurance from professional societies, even in late 2022, when women responded to our second questionnaire, concerns lingered. Also, vaccination-related changes to menstrual cycling, if present, are usually minimal (change in cycle length: 0.7 days), transient (<2 cycles) [20], and not unique to COVID-19 vaccination. Fertility (female or male) is not negatively affected [11]. Nonetheless, widespread press and social media coverage around these issues caused personal and public health harm, as demonstrated by increasing proportions of unvaccinated pregnant women in critical care units as 2021 progressed [21].

That the majority of those unvaccinated cited concerns about long-term side effects and lack of evidence regarding this raises an interesting contrast with prior vaccines. For pregnancy specifically, influenza and pertussis vaccination were recommended for use without such information, with limited evidence for reassurance even a decade later [22]. Long-term side effects appear to be a particular concern with COVID-19 vaccines. For example, the first COVID-19 vaccine was marketed in December 2020, and as of 14 November 2023, there were 197 PubMed citations about long-term side effects, whereas the influenza vaccine marketed in 1946 had 215 such citations. Also, there appeared to be a “nocebo” effect (expectation of a negative effect on health outcomes) associated with COVID-19 vaccination [23]. 

Acceptance of COVID-19 vaccination by pregnant or postpartum women in the U.K. has peaked at ≈75% [12]. Our cohort showed little vaccine delay, irrespective of pregnancy/postpartum status. Nonetheless, our responders’ free-text comments highlight struggles with misinformation and concerns about adverse effects that may have prompted others to hesitate or decline vaccination.

Communication interventions have varying success rates [24]. Greater success is seen with honest communication of the benefit–risk balance, use of humour, and presentation of vaccination as the social norm rather than the direct confrontation of scepticism (even when based on misinformation) [25]. These approaches have the potential to address concerns expressed by minoritised groups and improve their uptake of COVID-19 vaccination in the U.K. [9]. 

The World Health Organisation highlighted the impact of the “infodemic” accompanying the pandemic, which eroded trust in appropriate knowledge resources, unnecessarily confused and complexified personal decision making, and adversely affected public health strategies internationally [26]. Our responders’ views reflect these issues, with consequent personal distress. Our data show that healthcare workers are vulnerable to these same issues [27], reducing their capacity to provide timely and accurate vaccine advice. Our data emphasise the need for future consideration of these public health issues during vaccination campaigns, especially healthcare worker training [28].

### 4.3. Strengths and Limitations

The strengths of our study are its national (U.K.) recruitment via a freely available app that, at peak use during the pandemic, reached over 4 million individuals, mainly WRA [14]. Online participation minimised disruption to volunteers (many with young children), potentially contributing to suitable retention for the second questionnaire (61.7%). Staggered longitudinal questionnaire administration (separated by approximately nine months) ensured that women recruited when pregnant could ultimately consider and reflect upon their entire pandemic pregnancy experience. Our mixed-methods design allowed quantitative and qualitative data assessment, cross-sectionally and longitudinally. Our novel findings allow the opportunity for reflection upon the U.K. vaccination campaign and highlight a paradox not immediately evident from the prompt vaccination uptake by women of reproductive age; the success of the campaign was despite reservations expressed by women of reproductive age and required complex and very personal decision making. We believe these insights provide useful context for future vaccination campaigns, given the importance of vaccination both individually and collectively.

We considered whether study participation may be “triggering” for women with prior tragic pregnancies (e.g., stillbirth); however, these women now routinely contribute to relevant research [29], and some find exclusion from such research distressing and dismissive of their experience.

We readily acknowledge the limitations of our dataset. Our study population was not diverse, reflecting the general demographic of ZOE app users [14]; thus, our findings are limited in their generalizability within the U.K. and internationally. Also, it is difficult to gauge our response rates. Although the numbers of WRA within the COVID ZOE Study and the CSSB were known, the numbers of women considering pregnancy, pregnant, or recently pregnant could not be determined. Thus, our ability to assess for participation bias (including by reproductive status and vaccination type) is constrained; accordingly, our analyses are limited to descriptive statistics, and we do not draw associations from our data. Nevertheless, it is reassuring that the characteristics of responders and non-responders from among ZOE app users were similar (Table S4), with the exception that our cohort included a greater proportion of healthcare workers, and our recruitment rates reflected results from a previous study that suggested ≈3% of women KCL-CSS/ZOE app users aged 18–44 years were pregnant [30]. Lastly, in common with all studies requiring individual-level voluntary participation, our study will have a volunteer bias. 

With respect to our qualitative data, nearly a quarter of our cohort (852 of 3568 women) provided comments; however, only 167 (fewer than 5%) provided comments specific to vaccination experience, most of whom had experienced a pandemic pregnancy. Per our methods, providing questionnaire comments was optional, although open to all. It is plausible that some women might be more likely to provide comments than others—for example, women who experienced or attributed adverse vaccination outcomes. It is also possible that the views of women experiencing pandemic pregnancy might not reflect concerns of the cohort overall—for example, a primiparous woman contemplating vaccination mid-pregnancy might have differing concerns to those of a woman with an early pandemic pregnancy whose young child/ren could not access vaccination routinely. Neither possibility can be tested in our dataset. Also, we have not performed subtype analysis according to vaccination type due to small numbers; media coverage of side-effect profiles at this time was extensive and not uniform amongst preparations.

Given these potential biases, we would caution that our data cannot be used to draw conclusions regarding vaccination safety (including upon reproductive outcomes), which are better determined by randomised controlled trials along with post-marketing population monitoring (for example, as conducted routinely by the U.K. government’s Medicines and Healthcare Products Regulatory Agency).

## 5. Conclusions

Vaccines are critical for protecting public and personal health, including pregnancy and postpartum, for mothers and babies. Generally, our study participants were early adopters of COVID-19 vaccination and displayed altruistic and scientifically appropriate decision making. However, their free-text comments highlight the courage required to do this; they chose vaccination despite doubts and concerns. Additionally, it is important to note the deep personal responsibility women displayed in their decision making in an environment of unclear health information and vacillating guidance, which also affected health workers’ ability to provide robust and consistent vaccination counselling and advocacy. Vaccine acceptance should be nurtured to minimise reluctance to accept COVID-19 and other vaccinations over time.

## Figures and Tables

**Table 1:** Baseline characteristics of the women of reproductive age responders to the first and second surveys (N (%) or mean ± SD unless otherwise specified).

Characteristics	Total Consented Cohort (N = 3568)	Women Completing the FIRST Survey (N = 3453)	Women Completing the SECOND Survey (N = 2129) *
**Demographics**			
Age (years)	36.6 ± 5.0	36.6 ± 5.0	36.3 ± 4.8
Body mass index (kg/m^2^) Ł	25.6 ± 5.6	25.6 ± 5.6	25.3 ± 5.4
Weight (kg)	70.7 ± 16.4	70.7 ± 16.4	70.0 ± 15.8
Height (cm)	166.1 ± 6.6	166.1 ± 6.6	166.3 ± 6.7
Ethnicity			
White	3390 (95.0%)	3280 (95.0%)	2023 (95.0%)
Black or Black British	5 (0.1%)	4 (0.1%)	4 (0.2%)
Asian or Asian British	45 (1.3%)	43 (1.2%)	24 (1.1%)
Mixed/multiple	85 (2.4%)	85 (2.5%)	49 (2.3%)
Any other	29 (0.8%)	27 (0.8%)	13 (0.6%)
Not stated	14 (0.4%)	14 (0.4%)	6 (0.3%)
IMD (quintile)			
1 (most deprived)	273 (7.7%)	264 (7.6%)	161 (7.6%)
2	562 (15.8%)	540 (15.6%)	351 (16.5%)
3	746 (20.9%)	722 (20.9%)	439 (20.6%)
4	841 (23.6%)	814 (23.6%)	499 (23.4%)
5 (least deprived)	1042 (29.2%)	1011 (29.3%)	613 (28.8%)
*Missing*	104 (2.9%)		
**Healthcare worker**		707 (20.5%)	456 (21.4%)
**Past history**			
Health problems requiring you to stay home		144 (4.2%)	49 (2.3%)
High BP unrelated to pregnancy		72 (2.1%)	41 (1.9%)
Diabetes		54 (1.6%)	26 (1.2%)
Heart disease		16 (0.5%)	8 (0.4%)
Asthma or other lung disease		508 (14.7%)	284 (13.3%)
Kidney disease		7 (0.2%)	4 (0.2%)
Current smoker		71 (2.1%)	41 (1.9%)
Taking immunosuppressants		127 (3.7%)	76 (3.6%)
**Prior SARS-CoV-2 infection ǂ**			
None		1962 (56.8%)	410 (19.3%)
Confirmed		624 (18.1%)	1612 (75.7%)
More than once		34 (1.0%)	329 (15.5%)
Hospitalised (with COVID-19)		16 (0.5%)	12 (0.6%)
**Mental health status**			
Prior mental health condition		1135 (32.9%)	674 (31.7%)
Current PHQ2 score ≥3 ǁ		265 (7.7%)	174 (8.2%)
Current GAD2 score ≥3 ¶		575 (16.7%)	337 (15.8%)
**At least one prior pregnancy**		2277 (65.9%)	1576 (74.0%)
At least one miscarriage		913/2277 (40.1%)	682/1576 (43.3%)
At least one elective termination		362/2277 (15.9%)	213/1576 (13.5%)
At least one ectopic		72/2277 (3.2%)	46/1576 (2.9%)
Parous		2021/2277 (88.8%%)	1543/1576 (97.9%)
At least one stillborn baby		26/2277 (1.1%)	22/1576 (1.4%)
At least one liveborn baby		2018/2277 (88.6%)	1542/1576 (97.8%)
**Current pregnancy status**			
*Missing*		0	11 (0.5%)
Pregnant		524 (15.2%)	212 (10.0%)
Postpartum		72 (2.1%)	39 (1.8%)
Not pregnant or postpartum		2857 (82.7%)	1867 (87.7%)
Never pregnant		921 (26.7%)	433 (20.3%)
Pregnant in past (including earlier in pandemic)		1936 (56.1%)	1437/1867 (67.5%)
Trying to become pregnant **		516/2857 (18.1%)	281 (13.2%)

BP (blood pressure), GAD2 (Generalised Anxiety Disorder 2-item), IMD (index of multiple deprivation), PHQ2 (Patient Health Questionnaire-2). * Responders to the second survey include 36 individuals who responded only to the second survey. Ł Women may have been pregnant when reporting BMI. ǂ Note that the number of individuals with no SARS-CoV-2 infection and confirmed COVID-19 do not add to the total cohort due to difficulties accessing testing for SARS-CoV-2 at various times during the pandemic. ǁ A PHQ2 score ≥3 makes it likely a major depressive disorder. ¶ A GAD2 score ≥3 makes a generalised anxiety disorder possible and should prompt further evaluation. ** Women planning pregnancy may have been pregnant in the past or never pregnant.

**Table 2:** COVID-19 vaccine doses reported by women of reproductive age who responded to either survey.

Dose	FIRST Survey Responders (N = 3453)	Vaccine Type				SECOND Survey Responders (N = 2129)	Vaccine Type			
		Pfizer	Moderna	AZD	Other or NS *		Pfizer	Moderna	AZD	Other or NS *
0	61 (1.8%)	-	-	-	-	21 (1.0%)	-	-	-	-
1	3392 (98.2%)	2054 (60.6%)	186 (5.5%)	1123 (33.1%)	29 (0.9%)	2108 (99.0%)	1319 (62.6%)	116 (5.5%)	653 (31.0%)	20 (0.9%)
2	3379 (97.9%)	2063 (61.1%)	187 (5.5%)	1041 (30.8%)	88 (2.6%)	2106 (98.9%)	1326 (63.0%)	120 (5.7%)	610 (29.0%)	50 (2.4%)
3	3135 (90.8%)	2136 (68.1%)	820 (26.2%)	4 (0.1%)	175 (5.6%)	2056 (96.6%)	1353 (65.8%)	538 (26.2%)	1 (<0.1%)	164 (8.0%)
4	-	-	-	-	-	83 (3.9%)	27 (32.5%)	11 (13.3%)	-	45 (54.2%)
5	-	-	-	-	-	18 (0.8%)	7 (38.9%)	3 (16.7%)	-	8 (44.4%)
6	-	-	-	-	-	3 (0.1%)	2 (66.7%)	-	-	1 (33.3%)

AZD (Astra-Zeneca), NS (not stated). * For responders to the first survey, other vaccines specified for the first dose were J-J (N = 1) and Novavax (N = 1), and for the second dose, J-J (N = 1). No other vaccines were specified. For responders to the second survey, other vaccines specified were only for the first dose (i.e., Novavax, N = 1). No other vaccines were otherwise specified.

**Table 3:** Reasons for accepting or declining vaccination against SARS-CoV-2 *.

Reasons for Accepting/Declining Offer of Vaccination against SARS-CoV-2	FIRST Survey Responders (N = 3453)		SECOND Survey Responders (N = 2129) Ł	
	Vaccinated (N = 3392)	Not Vaccinated (N = 61)	Further Vaccinated * (N = 2090)	Remained Unvaccinated (N = 20)
**Overall benefits and risks**				
Think benefits of vaccination outweigh risks	3095 (89.6%)	NA	1846 (88.3%)	NA
I don’t feel the evidence of benefit is reliable	NA	15 (24.5%)	NA	8 (40.0%)
**Offer of vaccination**				
For my job	9454 (27.3%)	NA	492 (23.5%)	NA
Government recommends it	698 (20.2%)	NA	395 (18.9%)	NA
Received invitation from NHS/GP	1366 (39.6%)	NA	707 (33.8%)	NA
**Concern related to getting COVID-19**				
Worried about getting COVID-19	2300 (66.6%)	NA	1210 (57.9%)	NA
Worried about getting seriously ill from COVID	2399 (69.5%)	NA	1398 (66.9%)	NA
Have illness/medication that makes me more vulnerable to COVID	411 (11.9%)	NA	232 (11.1%)	NA
Have illness/medication that concerns me about vaccination	NA	9 (14.8%)	NA	0
Health condition that means unable to have vaccination/booster	NA	0	NA	0
Have had a family/friend who was very sick or who died from COVID	621 (18.0%)		315 (15.1%)	
Not concerned about getting COVID-19	NA	8 (13.1%)	NA	2 (10%)
Do not think at sufficient risk of getting COVID-19	NA	16 (26.2%)	NA	7 (35.0%)
Have had COVID	490 (14.2%)	NA	453 (21.7%)	NA
Think that they are immune	NA	22 (36.1%)	NA	11 (55.0%)
Natural (infection) is better	NA	16 (26.2%)	NA	5 (25.0%)
**Social activity**				
Yes (want to be active in community)	1967 (57.0%)	NA	951 (45.5%)	NA
Responsibility as member of community	2909 (84.2%)		1685 (80.6%)	
There is benefit if most people are vaccinated	3076 (89.1%)		1847 (88.4%)	
Worried about spreading COVID-19 to others	3050 (88.3%)		1776 (85.0%)	
Want to travel abroad again	988 (28.6%)		522 (25.0%)	
**Possible risks of COVID-19 vaccination**				
Risks very small	2531 (73.3%)		1608 (76.9%)	
Risks unacceptable	NA	17 (27.9%)	NA	12 (60.0%)
Concerned about vaccine adverse reaction	NA	2 (3.3%)	NA	12 (60.0%)
Concerned about vaccine long-term side effects	NA	41 (67.2%)	NA	16 (80.0%)
Concerned about development and approvals process	NA	13 (21.3%)	NA	7 (35.0%)
Do not know enough about vaccine	NA	17 (27.9%)	NA	10 (50.0%)
Concerned about the number of vaccine boosters needed	NA	NA	NA	2 (10%)
Do not think vaccine will work	NA	0	NA	1 (5.0%)
Do not think it will be available	NA	0	NA	0
**General views on vaccination**				
Support vaccination in general	3000 (86.9%)		1854 (88.7%)	
Against all vaccination	NA	0	NA	0
Religious reasons	0	1 (1.6%)	0	0
Personal belief/philosophical reasons	0	10 (16.4%)	0	13 (65.0%)
Other	108 (3.1%)	0	41 (2.0%)	0
**Pregnancy**				
Planning pregnancy	0	19 (31.1%)	0	7 (35.0%)
Receiving fertility treatment	0	5 (8.2%)	0	2 (10.0%)
Vaccine may affect pregnancy ǂ	NA	26 (42.6%)	NA	12 (60.0%)
Pregnant, wanted to keep me and my baby safe	535 (15.8%)	0	383 (18.3%)	0
Vaccine may not be safe during breastfeeding	NA	6 (9.8%)	NA	6 (30.0%)
Doctor/midwife advised against it or not able to have vaccinations	NA	2 (3.3%) ǁ	NA	2 (10.0%) ǁ

* Responses are not mutually exclusive, as women were asked to provide as many or as few reasons as applicable. Ł Of 2129 second survey responders, reasons for vaccination choice were provided by 2090 women who had accepted further vaccination since the first survey or who had received at least one vaccination having previously been unvaccinated, and by 20 women who remained unvaccinated. No further vaccination information was available for 15 women, and no reasons were provided by four women who were vaccinated only prior to the first survey with no subsequent vaccination. ǂ Whether the concern was related to maternal or fetal risk was not specified. ǁ Note that the qualitative data included many comments regarding healthcare professionals either ambivalent or advising against vaccination.

**Table 4:** Time to first vaccination, based on status at time of eligibility or at the time of vaccination.

	N	N (%) Vaccinated within 28 Days of Eligibility	Median Time from Date of Eligibility to Date of Vaccination [IQR]
**Status at time of eligibility for vaccination**			
All responders	3504 *	3278 (93.6%)	−7 [−75, +8]
General community	2689 Ł	2497 (92.85%)	+1 [−43, +9]
Women with at least one pregnancy during the pandemic	1352	1200 (88.75%)	+3 [−19, +12]
Women who were pregnant or postpartum at time of vaccine eligibility	642	580 (90.3%)	+1 [−38, +10]
Women who were pregnant	525	465 (88.6%)	+2 [−33, +11]
Women who were postpartum	117	115 (98.3%)	0 [−56, +6]
**Status at time of actual vaccination ǂ**			
Women who were pregnant or postpartum at time of vaccination	615	546 (88.8%)	+3 [−12, +12] ǁ
Women who were pregnant	512	454 (88.7%)	+3 [−18, +12]
Women who were postpartum	103	92 (89.3%)	+4 [−1, +10.5]
Women who were neither pregnant/postpartum when vaccinated	1890	1786 (94.5%)	0 [−52, +8] ǁ

IQR (interquartile range of 25th to 75th centiles). * Responders who could be assessed are all consented responders (n = 3568), excluding unvaccinated women (N = 44), women who received vaccination prior to official rollout (e.g., trial participants) (N = 14), and vaccinated women who had missing vaccination dates (N = 6). Note that as per study consent, vaccination data may have been available via the ZOE app for those participants whose CSSB first questionnaire data were incomplete. Ł The general community is defined as those who did not have early access to COVID-19 vaccination because of healthcare worker status (N = 711) and/or medical co-morbidities that mandated shielding (N = 145); these exclusion criteria are not mutually exclusive. ǂ This population excluded unvaccinated women [n = 44], women vaccinated prior to community rollout [n = 14], vaccinated women who were missing vaccination dates [n = 6], healthcare workers [n = 711], isolating individuals [n = 145], and women with pandemic pregnancy but missing expected or actual date of birth for baby [n = 235]); these exclusion criteria are not mutually exclusive. ǁ Median time from eligibility to vaccination was longer among women who were pregnant or postpartum when vaccinated, compared with those who were neither pregnant nor postpartum (*p*-value < 0.0001 [Z = −6.293] by approximative Wilcoxon–Mann–Whitney test, accounting for non-normal distribution).

**Table 5:** Key themes illustrated by 167 responders who made 171 comments, considered according to vaccine delay and pregnancy status (across the entire pandemic) *.

Reproductive Status	Quotations	
	Hesitant	NOT Hesitant
THEME 1: Strong motivation for vaccination (90 women, 92 comments)		
Pandemic pregnancy (82 women, 84 comments)	9 women (10 comments) “In my third trimester, had the community midwife been able to offer me the vaccine I would have taken her up on it. I felt poorly both mentally and physically throughout the pregnancy and therefore not as proactive as I should have been once the advice changed to strongly recommending the vaccine in pregnancy”.	73 women (74 comments) “I had to fight to get the vaccine, I was refused it first time, then rebooked as soon as government guidance changed. On my second visit, it took 2 h for the nurses to call a doctor, who then consented to me getting the vaccine, and then emailed through the forms. I was very keen to have the vaccine but I think others may have given up. I looked at data from the US who were encouraging vaccinations in pregnant women at the time, as well as RCOG guidance”. “I was vaccinated after giving birth but while still breastfeeding. When deciding to be vaccinated, the literature was pretty clear about the risks, but the doctors/medics involved in vaccination were very reluctant to discuss it. I think they were worried about liability for advice when breastfeeding… Thankfully, my husband and I had discussed it extensively and I was sure it was the right decision for me, my baby, and society at large, but if I hadn’t been assertive and sure, his reaction would have put me off having the second jab and also potentially make me regret the first”. “I was in my third trimester when it was officially stated that pregnant women could and should be vaccinated and were at increased risk. No health professionals at all mentioned they vaccine to me and midwives had no info or advice when I brought it up. It was only because…my MP [Member of Parliament] had arranged for pregnant women to receive excess Pfizer vaccines that I was able to get vaccinated, despite working in a face to face role and having to commute by public transport for work”.
Pre-pandemic pregnancy only (4 women, 4 comments)	(None)	4 women (4 comments) “I got vaccinated when still breastfeeding a 1 year old when the government/nhs/jcvi were still being overcautious so worried they would ask me and turn me away at the vaccine clinic if I was truthful. I knew logically that having a bit of non-living virus in breast milk would be better for both me and baby than catching the real virus that was common and killing people in the early days”.
Never pregnant (4 women, 4 comments)	(None)	4 women (4 comments) “I have been going through IVF treatment, which has been stressful, and meant I have shielded during this time. I will be having an embryo transfer in October and am concerned about getting Covid during this time, especially as I work teaching in a university which feels unsafe since mask mandates etc were dropped. I will be very anxious about getting Covid if I do get pregnant. I hope I can have another booster if I get pregnant”.
**THEME 2: Adverse effects of COVID-19 vaccination (72 women, 73 comments)**		
Pandemic pregnancy (59 women, 60 comments)	15 women (15 comments) “I chose not to get the COVID-19 vaccination until a few days before I knew I would give birth (was scheduled for induction…) due to ongoing concerns about safety. I had been very keen to participate in one of the pregnancy vaccine trials, however prior infection was an exclusion criteria for all of them which made me ineligible, but also made me more concerned about getting the vaccine outside of the trial if it had been deemed unsuitable for study under trial conditions”.	44 women (45 comments) “Whilst I think pregnant women should be encouraged to get the vaccine, I think there needs to be a lot more research done into women’s and pregnant people’s issues and the impact of the vaccine on them. I am a huge believer in vaccines and always take them and my child will be getting all offered vaccines but even I found it difficult to decide whether to get the vaccine whilst pregnant. On balance I decided in favour of it as there was some evidence that the antibodies would pass through the placenta and I wanted my baby to be born with some protection. I had to do a lot of my own research into quite scientific research papers…Preganhg [pregnant] people and people with babies have been an after thought for the government at best during this pandemic”. “I support vaccination and so intend to get vaccinated in future. However, even as a clinical academic who is familiar with miscarriage statistics and the safety and efficacy data in pregnancy of vaccination, the doubt that perhaps the vaccine contributed to my miscarriage persists. I wonder how many others who have miscarried experience this and how to support with managing these doubts? “I fell pregnant with twins straight after my first COVID vaccine and always wondered if there is a link between the vaccine and getting twins”.
Pre-pandemic pregnancy only (11 women, 11 comments)	(None)	11 women (11 comments) “Menstrual cycle massively effected after each vaccine and after covid. Usually 28 day cycle exactly, after first vaccine—41 day cycle, after 2nd vaccine—16 day cycle, after covid 38 day cycle”.
Never pregnant (2 women, 2 comments)	(None)	2 women (2 comments) “Would only get vaccination when start trying for a baby. Nervous to get it when actually pregnant. Other than that, not sure there is much point as have had 3 shots + covid so seems unlikely I’d get covid seriously (unless another strain & vaccine is updated for that specific strain). I felt quite I’ll [ill] from the vaccines so won’t do it again if I don’t have a purpose to (ie just another booster shot)”.
**THEME 3: Misinformation, misleading information, or ever-changing information (52 women, 55 comments)**		
Pandemic pregnancy (49 women, 52 comments)	9 women (10 comments) “The flip-flopping of advice for pregnant women and the fact that most guidance was based on self-reported real-world data from the US rather than RCT data made it a very difficult and confusing time (even for someone who is a clinical trialist and very much understanding of the value and necessity of vaccines ordinarily)”.	40 women (42 comments) “I feel the Government sent out confusing messages about the vaccine during my pregnancy”. “The promotion to pregnant women and information was very poor. I had a friend who went for her first vaccination and the volunteer administering it said ‘I hope your baby will be okay’. The mixed messaging resulted in anxiety for myself and many other mothers I know”. “I had very conflicting information about getting the Covid vaccine when I was pregnant. One midwife told me I shouldn’t get it, another said I should. It was the same when breastfeeding. I decided to get the vaccine after doing lots of my own research and in the hope that the baby would get some immunity too”. “I personally had no reservations in getting the vaccine once I understood that thousands of pregant people had been given Pfizer in the US with no ill effect, and that the vaccine is of of similar type to other vaccines (e.g flu) routinely given to pregnant people. However I think the mixed messages in the early days of the vaccine from the government, media and even sometimes healthcare professionals has had a huge detrimental effect and is the reason why many of the people hospitalised with covid at the moment are pregnant and unvaccinated. I also do not understand why the government seemingly ignored the increased risk to pregnant women in the third trimester, and did not prioritise pregnant people for vaccines. Every other higher risk group was prioritised, whereas pregnant people were told to be invited in line with their age group”.
Pre-pandemic pregnancy only (2 women, 2 comments)	(None)	Two women (2 comments) “I have had two failed rounds of IVF using donor eggs during this time. I had a 5-day embryo transfer on 22 December 2021, and tested positive for Covid on 27 December 2021. At this time, I had received two doses of vaccine, and was asked to delay the third due to proximity to embryo transfer and a lack of evidence as to potential impact. It is not possible to know whether a pregnancy would have resulted if I had not contracted Covid, or if I had been less ill due to having received the booster dose. A second embryo transfer in May 2022 was also unsuccessful”.
Never pregnant (1 woman, 1 comment)	(None)	1 woman (1 comment) “Pregnant women are eligible for boosters and it would be helpful if the government could clarify that this applies to those of us undergoing IVF too. Cycles are expensive and difficult and at risk of cancellation due to contracting covid—a booster would help put my mind at ease (I would continue to isolate etc.)”.
**THEME 4: Complicated decision making (51 women, 52 comments)**		
Pandemic pregnancy (46 women, 47 comments)	14 women (14 comments) “I waited to have my vaccine until after I had given birth as the advice I received was very confusing. A GP told me not to have it, then another one told me to read a lot of information and make a decision for myself. At 36 weeks I was advised to have it by a midwife, but it seemed too late so I waited. I think clear advice is needed on the risks and benefits and also advice on what to do if you choose not to be vaccinated eg mask wearing, avoiding crowded places etc”.	32 women (33 comments) “Advice about whether to get vaccinated while breastfeeding was very mixed and confusing and so is vaccination during pregnancy from what I have read. I was disappointed to find no one appeared to be noting if you were breastfeeding when you were vaccinated. I thought this was a missed opportunity to get valuable data”. “Being pregnant during the pandemic was very challenging. The decision to delay vaccination until baby was born was a very difficult risk to take. I still feel vulnerable to Covid and do not want to risk my newborn catching it”. “[I] paid for a private gp to remove my contraceptive implant as nhs were not providing this service. This private gp was the only person providing this service that I could find in the whole of northern england. I contacted the royal college of obstetricians and gynecologists and they basically told me that pregnancy should not be considered at this time (I can find the email if you want to read it). If that is the case, then why not tell EVERYONE not to get pregnant instead of just those on long term contraception? Because of this I am not returning to long term contraception as I want to control my own body”.
Pre-pandemic pregnancy only (2 women, 2 comments)	(None)	2 women (2 comments) “I was still breastfeeding when I had all my covid vaccinations and there wasn’t too much info available on the safety of the vaccine (or any long term data of course) so it was a slightly more stressful decision to make to be vaccinated than it would have otherwise have been”.
Never pregnant (3 women, 3 comments)	(None)	3 women (3 comments) “Would only get vaccination when start trying for a baby. Nervous to get it when actually pregnant. Other than that, not sure there is much point as have had 3 shots + covid so seems unlikely I’d get covid seriously (unless another strain & vaccine is updated for that specific strain). I felt quite I’ll from the vaccines so won’t do it again if I don’t have a purpose to (i.e., just another booster shot)”.

* Note that no unvaccinated individuals provided comments, and the theme categories are non-exclusive, so the summing of individual components exceeds the total number of participants and comments.

## Data Availability

Access to existing data or samples in the CSSB is through completion of a Data/Samples Access Request Form, submitted to css_research@kcl.ac.uk, and considered by the COVID Research Platform and Biobank Management Group, usually within two weeks of receipt. Costs may apply (See https://cssbiobank.com/information-for-researchers for details, accessed on 12 February 2024).

## Supplementary Materials

The following supporting information can be downloaded at: https://www.mdpi.com/article/10.3390/vaccines12040440/s1, Table S1: COVID Symptom Study Bank Group; Table S2: RESILIENT Study Group; Table S3: Vaccination eligibility according to UK governmental guidelines; Table S4: Demographic data of invited individuals (ZOE app users and existing CSSB members); and qualitative responders; Figure S1: Vaccination timing among responders, based on status at the time of eligibility for vaccination in the UK; Figure S2: Vaccination timing among responders, based on status at the time of actual vaccination.

## References

[1] Department of Health & Social Care Independent Report. Joint Committee on Vaccination and Immunisation: Advice on Priority Groups for COVID-19 Vaccination, 30 December 2020. Updated 6 January 2021. https://www.gov.uk/government/publications/priority-groups-for-coronavirus-covid-19-vaccination-advice-from-the-jcvi-30-december-2020/joint-committee-on-vaccination-and-immunisation-advice-on-priority-groups-for-covid-19-vaccination-30-december-2020#fn:3.

[2] Public Health England (2021). Press Release. JCVI Issues New Advice on COVID-19 Vaccination for Pregnant Women. https://www.gov.uk/government/news/jcvi-issues-new-advice-on-covid-19-vaccination-for-pregnant-women.

[3] Khalil A., Blakeway H., Samara A., O’Brien P. (2022). COVID-19 and stillbirth: Direct vs. indirect effect of the pandemic. Ultrasound Obs. Gynecol.

[4] Pérez-López F.R., Savirón-Cornudella R., Chedraui P., López-Baena M.T., Pérez-Roncero G., Sanz-Arenal A., Narváez-Salazar M., Dieste-Pérez P., Tajada M. (2022). Obstetric and perinatal outcomes of pregnancies with COVID 19: A systematic review and meta-analysis. J. Maternal-Fetal. Neonatal Med..

[5] Birol Ilter P., Prasad S., Mutlu M.A., Tekin A.B., O’Brien P., von Dadelszen P., Magee L.A., Tekin S., Tug N., Kalafat E. (2022). Maternal and perinatal outcomes of SARS-CoV-2 infection in unvaccinated pregnancies during Delta and Omicron waves. Ultrasound Obs. Gynecol..

[6] Gurol-Urganci I., Waite L., Webster K., Jardine J., Carroll F., Dunn G., Frémeaux A., Harris T., Hawdon J., Muller P. (2022). Obstetric interventions and pregnancy outcomes during the COVID-19 pandemic in England: A nationwide cohort study. PLoS Med..

[7] Royal College of Obstetricians and Gynaecologists, UK, Royal College of Midwives, UK Teratology Information Service, MacDonald Obstetric Medicine Society Information Sheet and Decision Aid: 24 March 2021. https://www.rcog.org.uk/globalassets/documents/guidelines/2021-02-24-combined-info-sheet-and-decision-aid.pdf.

[8] UK Health Security Agency (2021). COVID-19 Vaccine Surveillance Report. Week 4. https://assets.publishing.service.gov.uk/media/61f29e68d3bf7f78e2908eea/Vaccine-surveillance-report-week-4.pdf.

[9] Magee L.A., Molteni E., Bowyer V., Bone J.N., Boulding H., Khalil A., Mistry H.D., Poston L., Silverio S.A., Wolfe I. (2023). National surveillance data analysis of COVID-19 vaccine uptake in England by women of reproductive age. Nat. Commun..

[10] (2022). RCOG COVID-19 Vaccination Guidance Timeline. https://www.rcog.org.uk/media/k5aniwh1/rcog-covid-19-vaccination-guidance-timeline.pdf.

[11] (2023). COVID-19 Vaccines, Pregnancy and Breastfeeding FAQs. https://www.rcog.org.uk/guidance/coronavirus-covid-19-pregnancy-and-women-s-health/vaccination/covid-19-vaccines-pregnancy-and-breastfeeding-faqs/.

[12] UK Health Security Agency (2023). COVID-19 Vaccine Surveillance Report. Week 41. https://assets.publishing.service.gov.uk/media/6527f0bfaea2d0000d219c69/vaccine-surveillance-report-2023-week-41pdf.

[13] (2023). COVID-19 Vaccine Surveillance Report. Week 9. https://assets.publishing.service.gov.uk/government/uploads/system/uploads/attachment_data/file/1139990/vaccine-surveillance-report-2023-week-9.pdf.

[14] Menni C., Valdes A.M., Freidin M.B., Sudre C.H., Nguyen L.H., Drew D.A., Ganesh S., Varsavsky T., Cardoso M.J., El-Sayed Moustafa J.S. (2020). Real-time tracking of self-reported symptoms to predict potential COVID-19. Nat. Med..

[15] Hsieh H.-F., Shannon S.E. (2005). Three approaches to qualitative content analysis. Qual. Health Res..

[16] Press Release. Pregnant Women Urged to Come forward for COVID-19 Vaccination. UK Health Security Agency. 16 December 2021. https://www.gov.uk/government/news/pregnant-women-urged-to-come-forward-for-covid-19-vaccination#:~:text=We%20welcome%20the%20announcement%20from,are%20pleased%20they%20have%20listened.

[17] Poliquin V., Castillo E., Boucoiran I., Wong J., Watson H., Yudin M., Money D., Vanschalkwyk J., Elwood C., on behalf of the Infectious Disease Committee of the SOGC SOGC Statement on COVID-19 Vaccination in Pregnancy, 18 December 2020, Reaffirmed 12 March 2021. https://sogc.org/common/Uploaded%20files/Latest%20News/SOGC_Statement_COVID-19_Vaccination_in_Pregnancy.pdf.

[18] Wilcox A.J., Weinberg C.R., O’Connor J.F., Baird D.D., Schlatterer J.P., Canfield R.E., Armstrong E.G., Nisula B.C. (1988). Incidence of Early Loss of Pregnancy. N. Eng. J. Med..

[19] Rimmer M.P., Teh J.J., Mackenzie S.C., Al Wattar B.H. (2023). The risk of miscarriage following COVID-19 vaccination: A systematic review and meta-analysis. Hum. Reprod..

[20] Edelman A., Boniface E.R., Male V., Cameron S.T., Benhar E., Han L., Matteson K.A., Van Lamsweerde A., Pearson J.T., Darney B.G. (2022). Association between menstrual cycle length and COVID-19 vaccination: Global, retrospective cohort study of prospectively collected data. BMJ Med..

[21] (2021). One in Six Most Critically Ill NHS COVID Patients Are Unvaccinated Pregnant Women. https://www.theguardian.com/lifeandstyle/2021/oct/11/one-in-six-most-critically-ill-patients-are-unvaccinated-pregnant-women-with-covid.

[22] Fung S.G., Fakhraei R., Condran G., Regan A.K., Dimanlig-Cruz S., Ricci C., Foo D., Sarna M., Török E., Fell D.B. (2022). Neuropsychiatric outcomes in offspring after fetal exposure to maternal influenza infection during pregnancy: A systematic review. Reprod. Toxicol..

[23] Haas J.W., Bender F.L., Ballou S., Kelley J.M., Wilhelm M., Miller F.G., Rief W., Kaptchuk T.J. (2022). Frequency of Adverse Events in the Placebo Arms of COVID-19 Vaccine Trials: A Systematic Review and Meta-analysis. JAMA Netw. Open.

[24] Whitehead H.S., French C.E., Caldwell D.M., Letley L., Mounier-Jack S. (2023). A systematic review of communication interventions for countering vaccine misinformation. Vaccine.

[25] Wellcome Trust Report. https://wellcome.org/reports/effective-ways-increase-vaccination-rates-what-evidence-tells-us.

[26] Infodemic. https://www.who.int/health-topics/infodemic#tab=tab_1.

[27] Li M., Luo Y., Watson R., Zheng Y., Ren J., Tang J., Chen Y. (2021). Healthcare workers’ (HCWs) attitudes and related factors towards COVID-19 vaccination: A rapid systematic review. Postgrad. Med. J..

[28] Underwood T., Hopkins K.L., Sommers T., Howell C., Boehman N., Dockery M., Dubé A., Dhaliwal B.K., Kazi A.M., Limaye R. (2023). Shaping global vaccine acceptance with localized knowledge: A report from the inaugural VARN2022 conference. BMC Proc..

[29] Kim B.V., Aromataris E.C., Middleton P., Townsend R., Thangaratinam S., Duffy J.M.N., de Lint W., Coat S., Flenady V., Khalil A. (2021). Development of a core outcome set for interventions to prevent stillbirth. Aust. N. Zealand J. Obstet. Gynaecol..

[30] Molteni E., Astley C.M., Ma W., Sudre C.H., Magee L.A., Murray B., Fall T., Gomez M.F., Tsereteli N., Franks P.W. (2021). Symptoms and syndromes associated with SARS-CoV-2 infection and severity in pregnant women from two community cohorts. Sci. Rep..

