# Supplementary material for: Courage in Decision Making: A Mixed-Methods Study of COVID-19 Vaccine Uptake in Women of Reproductive Age in the U.K
Source: Vaccines (Basel). 2024 Apr 18;12(4):440. doi: 10.3390/vaccines12040440 (PMC11055058; doi:10.3390/vaccines12040440)
Supplement: Supplementary file 1 [file vaccines-12-00440-s001.zip › vaccines-2908351-supplementary.pdf]

SUPPLEMENTARY APPENDIX

	Title	Page number
**TABLES**		
S1	COVID Symptom Study Bank Group	2
S2	RESILIENT Study Group	4
S3	Vaccination eligibility according to UK governmental guidelines	5
S4	Demographic data of invited individuals (ZOE app users and existing CSSB members); and qualitative responders	6
**FIGURES**		
S1	Vaccination timing among responders, based on status at the time of eligibility for vaccination in the UK	7
S1a	Figure S1a: Vaccine timing in entire cohort	7
S1b	Figure S1b: Vaccine timing in the 2689 responders in the general community, excluding health care-workers (N=711) and those who were shielding (N=145)	7
S1c	Figure S1c: Vaccine timing among the 1352 women with at least one pregnancy during the pandemic	8
S1d	Figure S1d: Vaccine timing among women who were pregnant (N=525) or postpartum (N=117) during the pandemic at the time of vaccine eligibility	8
S2	Vaccination timing among responders, based on status at the time of actual vaccination	9
S2a	Vaccination timing among women who were pregnant (N=512) or postpartum (N=103) at time of first vaccination	9
S2b	Vaccination timing among women who were not pregnant or postpartum at the time of first vaccination	9
**METHODS**	Supplementary methods	10

Table S1: COVID Symptom Study Bank Group (with named authors shaded in grey)

Name	Position (role, organisation)
Michela Antonelli	Lecturer, Health Data Mining, School of Biomedical Engineering & Imaging Sciences, King's College London, London, UK
Vicky Bowyer	Senior Clinical Research Manager, Department of Twin Research and Genetic Epidemiology, King's College London, London, UK
Julia R. B. Brown	Research Assistant, Department of Twin Research and Genetic Epidemiology, King's College London, London, UK; Medical Student, James Cook University, Australia
Liane S. Canas	Postdoctoral Research Associate, School of Biomedical Engineering & Imaging Sciences, King's College London, London, UK
Joan Capdevila Pujol	Data Science Manager, Zoe Ltd
Nathan J. Cheetham	Senior Postdoctoral Data Scientist, Department of Twin Research and Genetic Epidemiology, King's College London, London, UK
Jie Deng	PhD student, School of Biomedical Engineering & Imaging Sciences, King's College London, London, UK
Katie J. Doores	Department of Infectious Diseases, King's College London, London, UK
Emma L Duncan	Professor of Clinical Endocrinology (KCL) and Honorary Consultant Physician (GSTT), Department of Twin Research and Genetic Epidemiology, King's College London, London, UK
Alexander Hammers	Professor of Imaging and Neuroscience (KCL), and Head of King's College London & Guy's and St Thomas' PET Centre, Deputy Director of the Centre for Medical Engineering, and Honorary Consultant Physician (GSTT), School of Biomedical Engineering & Imaging Sciences, King's College London, London, UK; King's College London & Guy's and St Thomas' PET Centre, King's College London, London, UK
Nicholas R. Harvey	Postdoctoral Research Associate, Department of Twin Research and Genetic Epidemiology, King's College London, London, UK
Christina Hu	Head of Product, Zoe Ltd
Eric Kerfoot	Postdoctoral Research Associate, School of Biomedical Engineering & Imaging Sciences, King's College London, London, UK
Michael H. Malim	Senior Vice Dean for Academic Strategy & Partnerships; Co-Director of the Programme of Infection & Immunity; Professor of Infectious Diseases, Department of Infectious Diseases, King's College London, London, UK
Marc Modat	Senior Lecturer, School of Biomedical Engineering & Imaging Sciences, King's College London, London, UK
Erika Molteni	Senior Lecturer, School of Biomedical Engineering & Imaging Sciences, King's College London, London, UK
Benjamin Murray	PhD student, School of Biomedical Engineering & Imaging Sciences, King's College London, London, UK
Marc F Österdahl	PhD student, Department of Twin Research and Genetic Epidemiology, King's College London, London, UK; Department of Ageing and Health, Guy's and St Thomas' NHS Foundation trust, London, UK
Sebastien Ourselin	Head of School, Biomedical Engineering & Imaging Sciences; Director, Wellcome/EPSRC Centre for Medical Engineering; Deputy Director, London Medical Imaging and Artificial Intelligence Centre for Value Based Healthcare, School of Biomedical Engineering & Imaging Sciences, King's College London, London, UK

Name	Position (role, organisation)
Tim D. Spector	Co-founder (ZOE); Professor of Genetic Epidemiology (KCL); and Honorary Consultant Physician (GSTT), ZOE Ltd, and Department of Twin Research and Genetic Epidemiology, King's College London, London, UK
Claire J Steves	Professor of Ageing and Health and Clinical Director of TwinsUK (KCL); Honorary Consultant Physician (GSTT), Department of Twin Research and Genetic Epidemiology, King's College London, London, UK; Department of Ageing and Health, Guy's and St Thomas' NHS Foundation trust, London, UK
Carole H. Sudre	Senior Research Fellow, MRC Unit for Lifelong Health and Ageing (UCL), School of Biomedical Engineering & Imaging Sciences, King's College London, London, UK; MRC Unit for Lifelong Health and Ageing, Department of Population Health Sciences, University College London, London, UK Centre for Medical Image Computing, Department of Computer Science, University College London, London, UK
Jonathan Wolf	Co-founder and CEO, Zoe Ltd

Table S2: RESILIENT Study Group (with named authors shaded in grey)

Member	Position (role, organisation)
**Chief Investigator**	
Professor Laura A. Magee	Professor of Women's Health, King's College London, UK
**Co-investigators**	
Professor Debra Bick	Professor of Clinical Trials in Maternal Health, Warwick Clinical Trials Unit, University of Warwick, UK
Dr Harriet Boulding	Senior Research Fellow, The Policy Institute at King's, King's College London, UK
Professor Peter von Dadelszen	Professor of Global Women's Health, King's College London, UK
Professor Emma L Duncan	Professor of Clinical Endocrinology, Twin Research & Genetic Epidemiology, King's College London, UK
Dr Abigail Easter	Senior Lecturer in Maternal & Newborn Health, King's College London, UK
Professor Julia Fox-Rushby	Professor of Health Economics, King's College London, UK
Professor Asma Khalil	Professor of Obstetrics and Maternal Fetal Medicine, St George's University Hospitals NHS Foundation Trust, London, UK
Dr Hiten D. Mistry	Senior Research Fellow, Department of Women and Children's Health, King's College London, UK
Professor Eugene Nelson	Professor of Community and Family Medicine, The Geisel School of Medicine at Dartmouth, The Dartmouth Institute, USA
Professor Lucilla Poston, CBE	Tommy's Charity Professor of Maternal & Fetal Health; Head, School of Life Course Sciences, King's College London, UK
Dr Paul Seed	Statistics Lead, Department of Women and Children's Health, King's College London, UK
Mr Sergio A. Silverio	Research Associate in Social Science of Women's Health, King's College London, UK
Dr Marina Soley-Bori	Research Fellow, Health economics, King's College London, UK
Ms. Aricca Van Citters	Senior Research Scientist, The Dartmouth Institute for Health Policy and Clinical Practice, Hanover, USA
Dr Sara White	Tommy's Maternal Diabetes Clinical Research Lead and Clinical Lecturer in Maternal Diabetes, King's College London, UK
Dr Ingrid Wolfe	Director, Institute of Women and Children's Health, King's College London, UK
**Collaborators**	
James MN Duffy	Specialist Registrar, Obstetrics and Gynaecology, North Middlesex Hospital, London, UK
Glyn Elwyn	Co-director of the Coproduction Laboratory within The Dartmouth Institute for Health Policy and Clinical Practice, Hanover, USA
Ken Hodson	Head of UK Teratology Information Service, UK
Nathalie MacDermott	Academic Clinical Lecturer, King's College London, UK
Rebecca Reynolds	Personal Chair of Metabolic Medicine, The University of Edinburgh, UK
Rachel Roberts	Research Manager, Tommy's Charity, UK

Table S3: Vaccination eligibility according to UK governmental guidelines [https://en.wikipedia.org/wiki/COVID-19_vaccination_in_the_United_Kingdom] https://www.gov.uk/government/publications/priority-groups-for-coronavirus-covid-19-vaccination-advice-from-the-jcvi-30-december-2020/joint-committee-on-vaccination-and-immunisation-advice-on-priority-groups-for-covid-19-vaccination-30-december-2020#fn3

Age/Demographic group defined by government	Age/Demographic group to avoid age overlaps	Date for vaccination access
Front-line health and social care workers	NA	Procedure set out on 9/1/2021 & 14/1/2021
Extremely clinically vulnerable group	NA	18/1/2021
70+ years	70+	18/1/2021
Shielding group	NA	15/2/2021
65-70 years	65-69 years	15/2/2021
60-65 years	60-64 years	1/3/2021
56-60 years	56-59 years	6/3/2021
50-56 years	50-55 years	17/3/2021
45-50 years	45-49 years	13/4/2021
44-45 years	44 years	26/4/2021
42-44 years	42-43 years	27/4/2021
40-42 years	40-41 years	30/4/2021
38-40 years	38-39years	13/5/2021
36-38 years	36-37 years	18/5/2021
34-36 years	34-35 years	20/5/2021
32-34 years	32-33 years	22/5/2021
30-32 years	30-31 years	26/5/2021
25-30 years	25-29 years	8/6/2021
23-25 years	23-24 years	15/6/2021
21-23 years	21-22 years	16/6/2021
18-20 years	18-20 years	18/6/2021

Table S4: Demographic data of invited participants (with some data from table 1 repeated here for ease of comparison).

Characteristics	**Invitees** (N=85,092)		**Responders to first survey** (N=3453) (as per Table 1)	**Responders only to second survey** (N=35)	**Responders providing qualitative comments in either survey** (N=852)
	ZOE app (N=82,622)	CSSB (N=3568)			
Age (years)	37.8 ± 6.4	36.6 +/- 5.0	37.6±5.0	36.5 +/- 4.2	36.7 +/- 3.9
BMI (kg/m^2)	26.1 ± 6.4	25.6 +/- 5.6	25.6±5.6	26.0 +/- 5.1	25.1 +/- 5.2
Ethnicity					
White	75,125 (90.9%)	3390 (95.0%)	3280 (95.0%)	34 (97.1%)	808 (94.8%)
Black or Black British	343 (0.4%)	5 (0.1%)	4 (0.1%)	-	1 (0.1%)
Asian or Asian British	1617 (2.0%)	45 (1.3%)	43 (1.2%)	1 (2.9%)	11 (1.3%)
Mixed or multiple ethnic groups	1745 (2.1%)	85 (2.4%)	85 (2.5%)	-	23 (2.7%)
Any other ethnic group	664 (0.8%)	29 (0.8%)	27 (0.8%)	-	4 (0.5%)
Not stated	3128 (3.8%)	14 (0.4%)	14 (0.4%)	-	5 (0.6%)
IMD					
1 (most deprived)	6292 (7.6%)	273 (7.6%)	264 (7.6%)	2 (5.7%)	56 (6.6%)
2	12,870 (15.6%)	562 (15.7%)	540 (15.6%)	7 (20.0%)	133 (15.6%)
3	16,321 (19.8%)	746 (20.9%)	722 (20.9%)	8 (22.9%)	183 (21.5%)
4	18,394 (22.3%)	841 (23.6%)	814 (23.6%)	7 (20.0%)	212 (24.9%)
5 (least deprived)	22,807 (27.6%)	1042 (29.2%)	1011 (29.3%)	9 (25.7%)	247 (29.0%)
Missing	5938 (7.2%)	104 (2.9%)	0	2 (5.7%)	21 (2.5%)
Healthcare worker	3066 (3.9%)	711 (19.9%)	707 (20.5%)	3 (8.6%)	173 (20.3%)
Vaccination	80,446 (97.4%)	3524 (98.7%)	3409 (98.7%)	35 (100%)	845 (99.2%)
Confirmed COVID	41,382 (50.1%)	2040 (57.2%)	624 (18.1%)	15 (42.9%)	559 (65.6%)
Missing	23 (0.03%)	32 (0.90%)	-	10 (28.6%)	2 (0.2%)

BMI (body mass index), CSSB (COVID Symptom Study Biobank), IMD (index of multiple deprivation)

Figure S1: Vaccination timing among responders, based on status at the time of eligibility for vaccination in the UK*
Legend:
**These data exclude unvaccinated women, vaccinated women with missing vaccination dates, and vaccinated women who had access earlier than community rollout.*
HCW (healthcare worker)
Figure S1a: Vaccine timing in entire cohort
Figure S1b: Vaccine timing in the 2689 responders in the general community, excluding health care-workers (N=711) and those who were shielding (N=145)
Figure S1c: Vaccine timing among the 1352 women with at least one pregnancy during the pandemic
Figure S1d: Vaccine timing among women who were pregnant (N=525) or postpartum (N=117) during the pandemic at the time of vaccine eligibility

Figure S1a

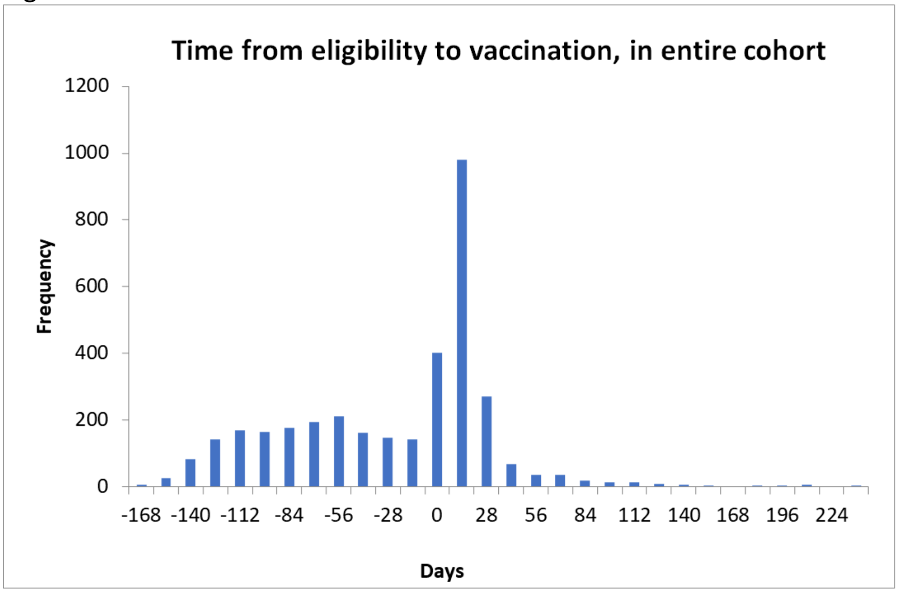

Figure S1b

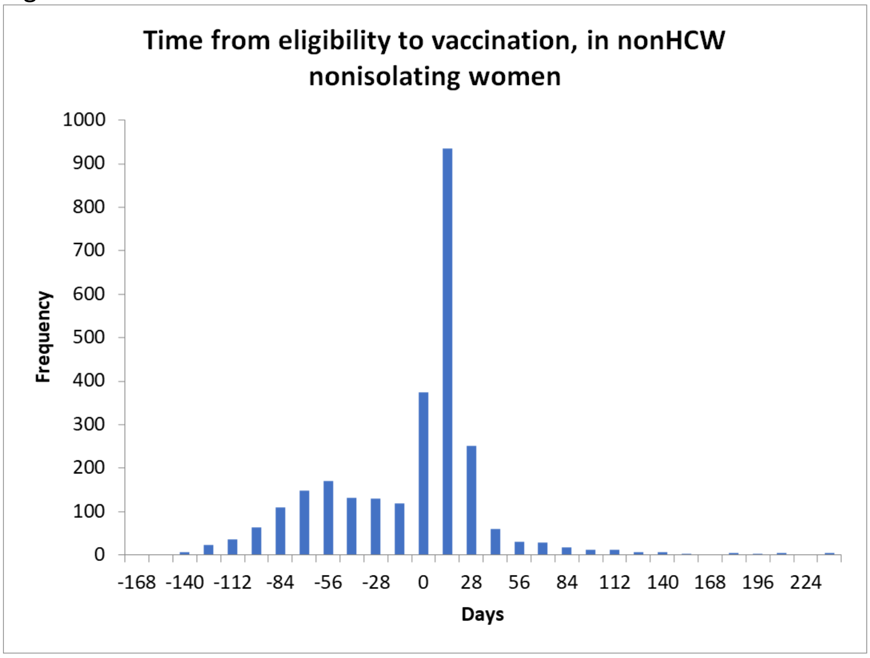

Figure S1c

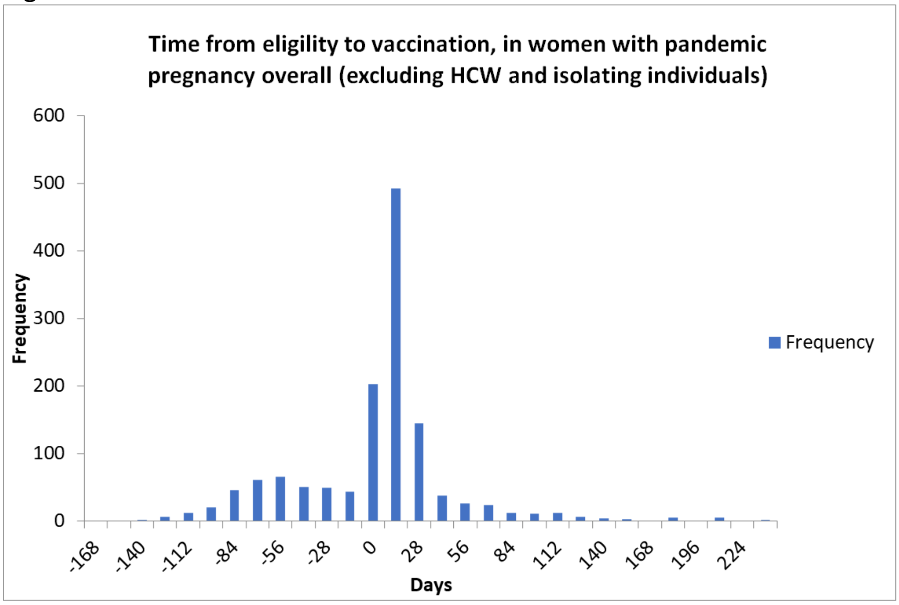

Figure S1d

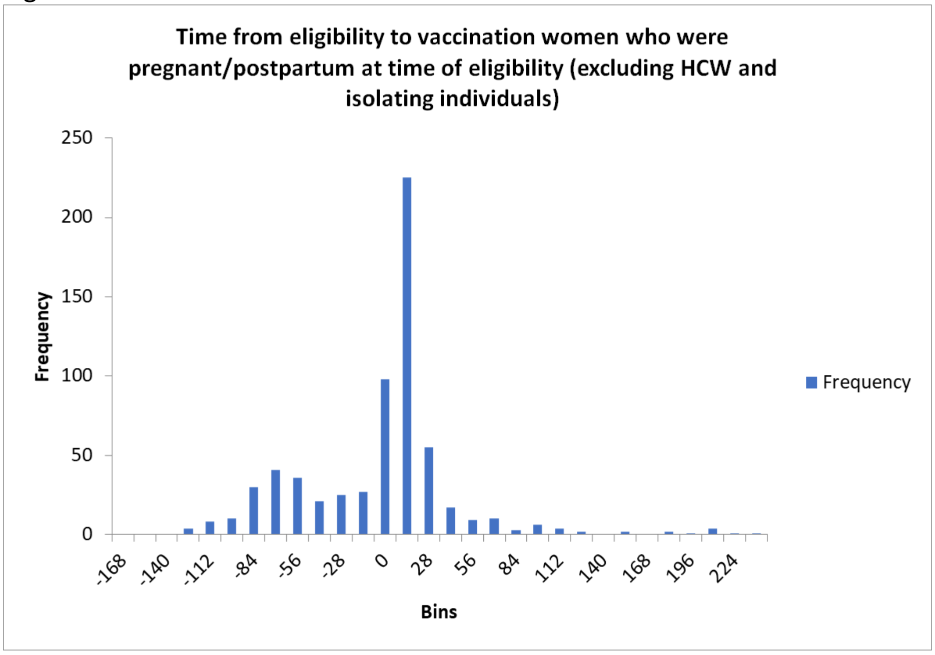

Figure S2: Vaccination timing among responders, based on status at the time of actual vaccination*
Legend:
*These data exclude unvaccinated women, vaccinated women with missing vaccination dates, and vaccinated women who had access earlier than community rollout. HCW (healthcare worker).
Figure S2a: Vaccination timing among women who were pregnant (N=512) or postpartum (N=103) at time of first vaccination
Figure S2b: Vaccination timing among women who were not pregnant or postpartum at the time of first vaccination

Figure S2a

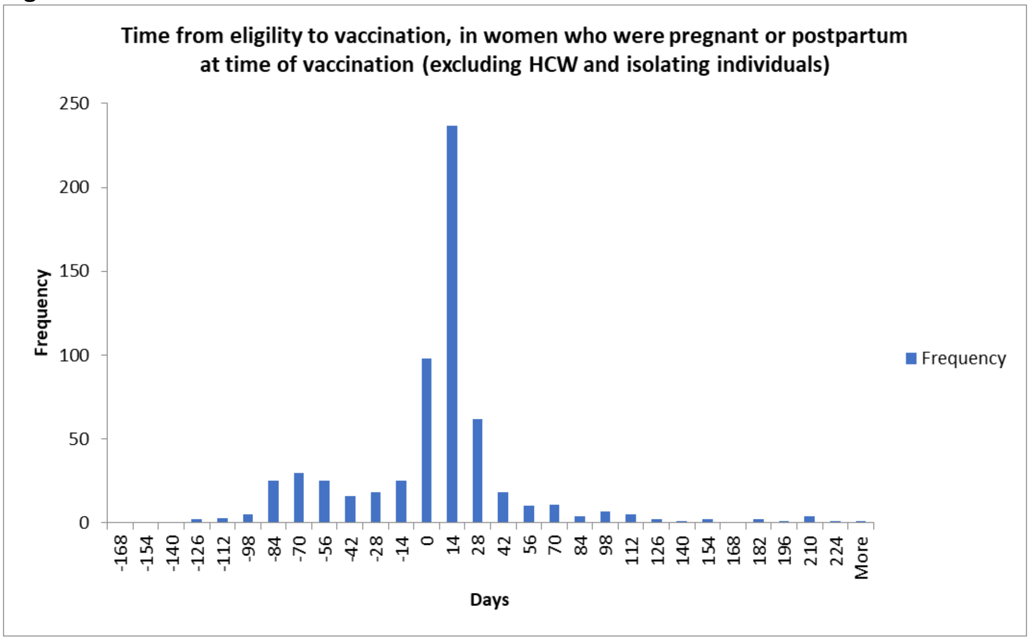

Figure S2b

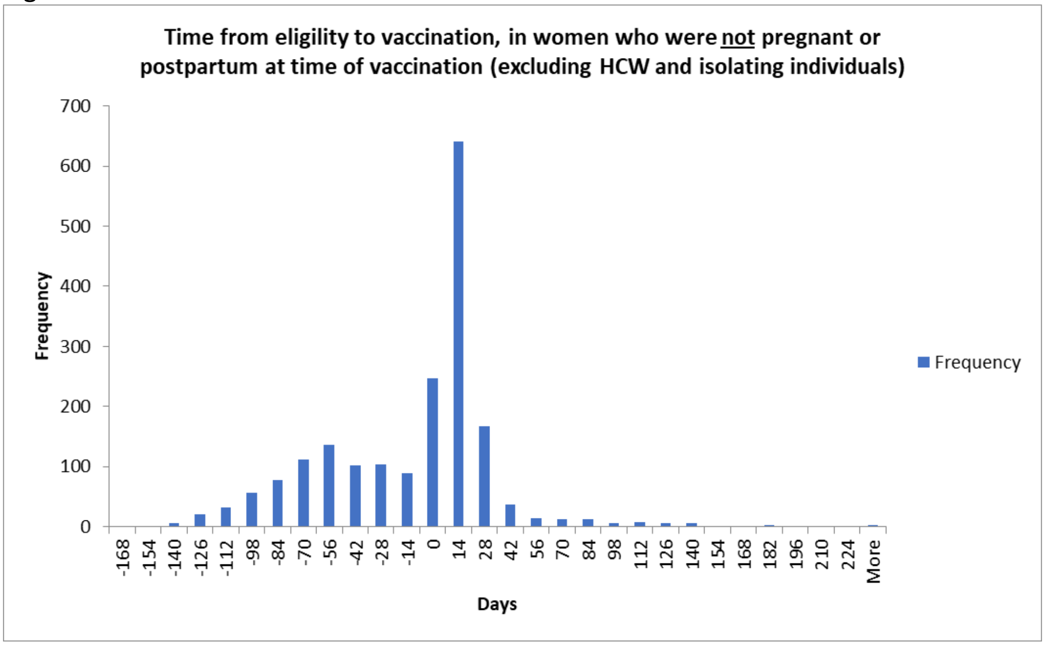

SUPPLEMENTARY METHODS

COVID Symptom Study / ZOE app

The KCL-CSS/ZOE app was launched jointly by ZOE Ltd. and King's College London (KCL) researchers on 24 March 2020, with researchers from the Massachusetts General Hospital, USA; and Swedish Universities Lund and Uppsala [14]. The COVID Symptom Study Biobank (CSSB) was established in September 2020, to support research into the impact and effects of COVID-19, particularly long illness duration, with independent ethics approval and governance structure.

With over six million users since its launch, most (>4.5 million) live in the UK. On registration, participants gave informed consent for their self-reported data to be used in COVID-19-related research, and provided basic demographic information (e.g., demographics, including self-identified ethnicity based on categories from the Office for National Statistics, UK[15]; education and income (based on English Indices of Deprivation 2019, IMD); healthcare worker status; and co-morbidities).[16] Subsequently, individuals were asked to log, on a daily basis, their symptoms, any SARS-CoV-2 testing, results, or care, and (once available) vaccination details. ZOE-app users could also be invited to participate in various sub-studies.

COVID Symptom Study Biobank

Individuals were recruited to CSSB from the ZOE app active user base, by direct invitation, according to illness duration and SARS-CoV-2 status, balanced by age and gender, but not ethnicity (noting that ZOE app users are over-representative of individuals from White British backgrounds, less deprivation, more educated, and more healthcare workers)[14]. Individuals in CSSB could participate in CSSB research independently of the KCL-CSS/ZOE study, whilst still fully participating in the app directly; and CSSB researchers could access ongoing data logged by CSSB participants via the ZOE app (e.g., vaccinations).

SUPPLEMENTARY APPENDIX – QUESTIONNAIRES

TITLE	PAGE NUMBER
Baseline questionnaire	2
Table 1: Alterations of baseline questionnaire for follow-up questionnaire	29

BASELINE QUESTIONNAIRE

- Blocks 2 to 8 were shown to all respondents.
- Block 9 was shown to respondents who reported one or more live births since February 2020.
- Blocks 11 and 13 were optional sections shown to respondents who reported one or more live births since February 2020 and who agreed to continue.
- The questionnaire was administered using Qualtrics (Qualtrics, Provo, UT) hosted at King's College London.

2. PREGNANCY STATUS

These first questions are about pregnancy and contemplating pregnancy.
Questions about pregnancy and pregnancy planning can be sensitive. If you find any of the questions upsetting, please accept our apologies. We ask these questions because they are important in helping us to understand how the pandemic has affected women of childbearing age.

2.2
Which of the following best describes your current pregnancy status?
- I have never been pregnant → 2.3
- I am not pregnant, but I have been in the past (including miscarriage, ectopic pregnancy, elective termination, stillbirths, and livebirths) → 2.3
- I am pregnant now → 2.6

	I have never been pregnant.	I am not pregnant but have been in the past.	I am pregnant now.
2.3<br>Are you thinking about becoming pregnant?<br>○ No<br>○ Yes	No → 2.7<br>Yes → 2.4		
2.4<br>Are you trying to become pregnant now?<br>○ No<br>○ Yes	No → 2.7<br>Yes → 2.5		
2.5<br>For how many months have you been trying?<br>Text entry: Number of months	→ 2.6		
2.6<br>Have you had treatment from a doctor to help you become pregnant?<br>○ No<br>○ Yes	→ 2.7		→ 2.8
2.7<br>Have your pregnancy plans changed at all because of the pandemic?<br>○ No<br>○ Yes	→ 3.1	→ 2.9	
2.8<br>What is your due date?<br>Enter date			→ 2.9
2.9<br>How many times have you ever been pregnant? Please include the current pregnancy if you are pregnant today and any previous miscarriages, elective terminations, ectopic pregnancies, stillborns, and liveborns.<br>*Enter the number in the box below.*<br>Enter number		→ 3.1 if answer is 0.	

2. PREGNANCY STATUS

2.10
Shown if 2.2 answer is I am pregnant now AND 2.9 ≥ 2, OR 2.2 answer is I am not pregnant now but have been in the past.
We would now like to ask you about your pregnancies.
Have you ever had any of the following?
Please enter the number of times below.

Miscarriage	
Elective termination	
Ectopic (tubal) pregnancy	
Stillborn baby	
Liveborn baby	
Total (calculated automatically)	

→ 2.11 if ≥ 1 for stillborn

→ 2.12

2.12 *Shown if 2.9 ≥ 2.*
How many times have you been pregnant since February 2020 (when the pandemic started in the UK)?
Enter the number of times.
Enter number

→ 3.1 (unless 2.12 for pregnancies is ≥1 AND 2.10 for liveborns ≥ 1)

2.13 *Shown if 2.12 for pregnancies is ≥1 AND 2.10 for liveborns ≥ 1.*
How many liveborn babies have you had since February 2020?
Enter the number in the box below.
Enter number

3. COVID VACCINATIONS

We'd now like to ask you about any COVID-19 vaccine injections you may have received.

3.2
Have you had a COVID-19 vaccination?
- No → 5.1
- Yes, via a national COVID-19 vaccination programme (e.g., NHS)
- Yes, but only as part of a clinical trial

3.3
How many COVID-19 vaccine doses have you had? *If you have only had a vaccine as part of a clinical trial, do not include any placebo (dummy vaccine) doses.*
Please enter the number of COVID-19 vaccinations in the box below.
Enter number

4. COVID VACCINATION HISTORY (DOSE LOOP)

Shown if response to 3.2 is Yes. Block repeats (loops) for the number of instances recorded in 3.3.
These questions are about your Nth vaccine dose.

4.2
What is the name of the Nth vaccine you received?
- Pfizer/BioNTech
- Oxford/AstraZeneca
- Moderna
- J&J/Janssen
- Novavax
- Other/don't know

4.3
What date did you have your Nth [pipe in vaccine name] injection? If you do not remember exactly, please put your best estimate.
Enter date

4.4

4. COVID VACCINATION HISTORY (DOSE LOOP)

In the hours to days following your vaccination, did you experience any symptoms near the injection site after your Nth injection [name of vaccine]? *Choose ALL that apply.*

- ☐ *Pain or tenderness*
- ☐ *Redness or warmth*
- ☐ *Swelling or bruising*
- ☐ *Swollen glands in armpit.*
- ☐ *Itch*
- ☐ *Other*
- ☐ *None*

4.5
Did you experience any other symptoms after your Nth injection? *Choose ALL that apply.*

- ☐ Feeling tired, achy or like being sick
- ☐ A headache → 4.6
- ☐ A rash that looked like small bruises or bleeding under the skin
- ☐ Shortness of breath, chest pain, leg swelling or persistent abdominal (tummy) pain
- ☐ Feeling hot or shivery, or had a fever → 4.7
- ☐ A new, continuous cough
- ☐ Loss or change to your sense of smell or taste
- ☐ None → 5.1

→ 4.8 unless otherwise stated

4.6
Was your headache like any of the following? *Choose ALL that apply.*

- ☐ Worse when you are lying down or bending over
- ☐ Severe and not relieved with painkillers
- ☐ Unusual for you and occurred with blurred vision, feeling or being sick, problems speaking, weakness, drowsiness or seizures (fits)
- ☐ None of the above

All answers → 4.8

4.7
Did your feeling of being hot or shivery, or having a fever last longer than two days?

- ○ No
- ○ Yes

4.8
Did you seek medical attention for any of these symptoms?

- ○ No → end of block or repeat block (if 3.3 >1 i.e., have received more than one vaccine dose)
- ○ Yes → 4.9

4.9
Please tell us what you were told.

Free text

→ end of block, or repeat of block (if 3.3 >1, i.e., have received more than one vaccine dose)

5. VIEWS ON VACCINATION

In this section, we would like to ask your views on vaccination.

5.2 *Shown if response to 3.2 is No*
Would you have a COVID-19 vaccine if offered?

- ○ No
- ○ Yes → 5.6

5.3
Please tell us why you have not had or would not have a COVID-19 vaccination, if offered. *Choose ALL that apply.*

- ☐ I took part in a vaccine trial
- ☐ I've had COVID and think I am now protected by antibodies (have immunity)
- ☐ Religious reasons
- ☐ Personal belief/philosophical reasons
- ☐ My doctor/midwife has advised against it, or I am not able to have vaccinations
- ☐ Planning pregnancy
- ☐ Receiving fertility treatment
- ☐ Concerned it may affect my pregnancy
- ☐ Concerned that it may not be safe during breastfeeding
- ☐ Concerned about the long-term side effects
- ☐ Do not know enough about it
- ☐ Illness/medication
- ☐ Do not think it will be available to me
- ☐ I do not think I am at sufficient risk
- ☐ I'm concerned about the development and approval process
- ☐ I feel that the possible risks are unacceptable
- ☐ Natural (infection) is better
- ☐ I'm not concerned about getting seriously ill
- ☐ I don't feel the evidence of benefit is reliable
- ☐ Do not think it will work
- ☐ Concerned about adverse reaction
- ☐ I am against all vaccination → 5.4
- ☐ Other → 5.5
- ☐ Prefer not to say

→ 5.8 (unless otherwise stated)

5.4 *Shown if response to 5.3 is 'I am against all vaccination'*
Please share your views with us about why you are against all vaccination.
Free text
→ 5.8

5.5 *Shown if response to 5.3 is 'Other'*
Please specify the other reason(s) why you would not have had a COVID-19 vaccination
Free text
→ 5.8

5.6 *Shown if response to 3.2 is Yes or 5.2 is Yes*
Why would or why did you have a COVID-19 vaccine? *Choose ALL that apply.*

- ☐ I am worried about getting COVID-19
- ☐ I am worried about getting really sick from COVID-19
- ☐ I have had COVID-19
- ☐ I have had a family/friend who was very sick or who died from COVID-19
- ☐ I am worried about spreading COVID-19 to others, including family and friends
- ☐ I think that the benefits outweigh the risks
- ☐ There is benefit if most people are vaccinated
- ☐ The risks to me of vaccination are very small
- ☐ I support vaccination when it is available
- ☐ I have an illness or take medication that makes me more vulnerable to COVID-19
- ☐ I am pregnant and want to keep me and my baby safe
- ☐ I think that it is my responsibility as a member of my community
- ☐ I want to be active in my community again, without fear of getting sick from COVID-19
- ☐ I received an invitation from the NHS/my GP
- ☐ To travel abroad
- ☐ For my job
- ☐ The government recommended it
- ☐ Other reason → 5.7
- ☐ Prefer not to answer

→ 5.8 unless otherwise stated

5.7 *Shown if response to 5.6 is 'Other'*
Please specify the other reason(s) why you would or did have a COVID-19 vaccination.
Free text

5.8
Do you think that pregnant or breastfeeding women should be recommended to receive the COVID-19 vaccine?
- No
- Yes
- Unsure
- Prefer not to say

3b. FLU VACCINATION

These questions are about flu vaccinations.

3b.1
Have you had a flu (influenza) vaccination this autumn/ winter (i.e., since 1st September 2021)?
- No → 3b.2
- Yes → 3b.3

3b.2 Shown if answer to 3b.1 is No.
Would you have a flu vaccine if offered this autumn/ winter?
- No
- Yes
- Not sure

3b.3 *Shown if answer to 3b.1 is Yes.*
What date did you have your flu vaccine. If you do not remember exactly, please put your best estimate.
Free text

6. COVID-19 HISTORY

These questions are about having COVID-19.

6.2
Do you think that you currently have or have ever had COVID-19?
- Yes, confirmed by a positive test
- Yes, based on medical advice
- Yes, based on strong personal suspicion
- Unsure → 7.1
- No → 7.1
- Prefer not to say → 7.1

6.3
Do you know the date when you first got (or might have got) COVID-19?
- I know the date (or the rough date) → 6.4
- Don't know → 6.5
- Prefer not to answer → 6.5

6.4 *Shown if response to 6.3 is 'I know the date'*
Please enter the date when you first got (or might have got) COVID-19. If you do not remember exactly, please put your best estimate.
Enter date

6.5
At the time of having or possibly having COVID-19, did you have symptoms of COVID-19, such as fever, cough, loss of taste or smell, or sneezing?
- No → 6.7
- Yes

6.6
Have you ever had to stay in hospital because of COVID-19 symptoms?

6. COVID-19 HISTORY

- Yes
- No
- Don't know
- Prefer not to answer

6.7
Do you think you have had COVID-19 more than once?
- Yes, confirmed by a second positive test
- Yes, based on medical advice
- Yes, based on strong personal suspicion
- Unsure → 7.1
- No → 7.1
- Prefer not to say → 7.1

6.8
Do you know the date when you got (or might have got) COVID-19 the second time?
- I know the date (or the rough date)
- Don't know → 6.10
- Prefer not to answer → 6.10

6.9 *Shown if response to 6.8 is 'I know the date'*
Please enter the date when you got (or might have got) COVID-19 the second time. If you do not remember exactly, please put your best estimate.
Enter date

6.10
How long have you had, or did you have COVID-19 symptoms overall? Please include time spent with mild symptoms and the time in between symptoms if these have been coming and going.
If you caught COVID more than once, please answer about the longest episode of illness you experienced.
- Up to 1 week (0 to 7 days)
- 1 to 2 weeks (8 to 14 days)
- 2 to 3 weeks
- 4 to 12 weeks
- More than 12 weeks

7. GENERAL & MENTAL HEALTH

Now we would like to ask some questions about your health in general.

7.2
In general, do you have any health problems that require you to stay at home?
- No
- Yes

7.3
Do you have any of the following health problems?

	No	Yes
High blood pressure (not related to pregnancy)		
Diabetes		→ 7.4
Heart disease		
Asthma or another lung disease		
Kidney disease		

7.4 *Shown if response to 7.3 is Yes*
What type of diabetes do you have?
- Type 1 diabetes
- Type 2 diabetes
- Gestational diabetes (that is, only during pregnancy)
- Unsure what type of diabetes

7.5

7. GENERAL & MENTAL HEALTH

Have you ever been told that you have cancer?

- ○ No → 7.7
- ○ Yes →7.6

7.6 *Shown if response to 7.5 is Yes*

What type(s) of cancer do you have now, or did you have in the past?

Free text

7.7 Are you using any of the following forms of contraception? *Choose ALL that apply.*

- ☐ Combined oral contraceptive pill or progesterone only pill
- ☐ Mirena or another hormone coil
- ☐ Depot injection or implant
- ☐ Condoms or other form of barrier contraception
- ☐ Other
- ☐ None
- ☐ Prefer not to say

7.8

Do you regularly take immunosuppressant medications (including steroids, methotrexate, or biologics)?

- ○ No
- ○ Yes

7.9

Do you smoke?

- ○ No, I have never smoked
- ○ Not now, but I have in the past
- ○ Yes, I currently smoke

The next questions are about your mental health.

7.11

Please read each item and select the reply which comes closest to how you have been feeling.

Don't take too long over your replies. Your immediate reaction to each item will probably be more accurate than a long thought-out response.

Over the last 2 weeks, HOW OFTEN have you been bothered by the following problems:

	Not at all	Several days	More than half the days	Nearly every day
Little interest or pleasure in doing things (PHQ1)				
Feeling down, depressed or hopeless (PHQ2)				
Feeling nervous, anxious or on edge (GAD1)				
Not being able to stop or control worrying (GAD2)				

7.12

Have you had any concerns about your mental health or emotional wellbeing since the beginning of the pandemic (in February 2020 in the UK)?

- ○ No
- ○ Yes

7.13

Have you ever been diagnosed with a mental health condition?

- ○ No
- ○ Yes

7. GENERAL & MENTAL HEALTH

- ○ Unsure
- ○ Prefer not to say

7.14 *Shown if response to 7.13 is Yes*

Please tell us which of the following you have been diagnosed with. *You can choose more than one.*

- ☐ Generalised anxiety disorder (GAD)
- ☐ Panic disorder
- ☐ Specific phobias
- ☐ Obsessive compulsive disorder (OCD)
- ☐ Post-traumatic stress disorder (PTSD), or complex PTSD
- ☐ Social anxiety disorder
- ☐ Agoraphobia
- ☐ Depression
- ☐ Attention deficit or attention deficit and hyperactivity disorder (ADD / ADHD)
- ☐ Eating disorder (e.g., bulimia nervosa; anorexia nervosa; psychological over-eating or binge-eating)
- ☐ Personality disorder
- ☐ Postpartum depression
- ☐ Postpartum psychosis
- ☐ Mania, hypomania, bipolar or manic depression
- ☐ Schizophrenia
- ☐ Substance use disorder
- ☐ Any other type of psychosis or psychotic illness
- ☐ Other
- ☐ Prefer not to say

8. RISK FACTORS FOR COVID-19

These questions are about risk factors for COVID infection.

8.2

Are you a health care worker (including hospital, elderly care or in the community)?

- ○ Yes, I currently interact with patients
- ○ Yes, but I do not currently interact patients
- ○ No → 8.5

8.3

Have you ever interacted (in person or face-to-face) with patients with confirmed or suspected COVID-19 infection?

- ○ Yes, confirmed COVID-19 cases only
- ○ Yes, suspected COVID-19 cases only
- ○ Yes, both confirmed & suspected COVID-19 cases
- ○ No, not that I know of

8.4

Since the COVID-19 pandemic began, have you used personal protective equipment (PPE) at work?
(Depending on your specific work requirements, PPE might include gloves, masks, face shields, etc.)

- ○ Always
- ○ Sometimes
- ○ Never

8.5

Have you EVER been exposed to someone with confirmed or suspected COVID-19 infection (such as co-workers, family members, or others)? *Choose ALL that apply.*

- ☐ Yes, confirmed COVID-19 cases
- ☐ Yes, both confirmed & suspected COVID-19 cases
- ☐ Yes, presumed COVID-19 cases
- ☐ No, not that I know of

8.6

Have you been self-isolating at all over the last week?
- No, not at all
- Completely - I have not left the house
- I have rarely left the house and when I have, I have had little interaction with others (e.g., for exercise)
- I have rarely left the house, but had to visit somewhere with lots of people (e.g., hospital/clinic, groceries)
- I have sometimes left the house, but I had little interaction with others
- I have sometimes left the house to visit somewhere with lots of people
- I have often left the house, but I had little interaction with others
- I have often left the house and was in contact with other people (e.g., still working outside the house or using public transport)

8.7
In the last week, did you wear a face mask (or other face covering) indoors when away from home (other than at work)? *Please choose ONE answer.*
- Never
- Sometimes
- Most of the time
- All of the time
- Not applicable

9. BABIES BORN SINCE FEBRUARY 2020 *Shown to all who have had ≥1 livebirth since February 2020*

These questions are about your liveborn baby(ies) born since February 2020. We will ask about each baby separately, including twins and triplets, in the order in which they were born.
The next questions are about [baby number] your Nth liveborn baby since February 2020.

9.3
Was [baby number] born from a single or multiple baby pregnancy?
- Single – only ONE baby
- Multiple – one of TWO babies (twins)
- Multiple – one of THREE babies (triplets)
- Multiple – one of FOUR babies (quadruplets)

9.4
Did you have any problems with the following during your pregnancy with [baby number]?
Please choose all options that are relevant and choose 'no' if you had none of the conditions listed. *Choose ALL that apply.*
- ☐ High blood pressure
- ☐ Gestational diabetes
- ☐ Urine infection
- ☐ Preterm birth
- ☐ Other → 9.5
- ☐ No

All answers to 9.6, unless otherwise stated

9.5 *Shown if answer to 9.4 is Other*
Please let us know about the other health problem(s) you had with [baby number].
Free text

9.6
How was [baby number] born?
- Vaginal birth with assistance (such as, forceps, vacuum, or breech delivery)
- Vaginal birth with no assistance
- Caesarean - emergency (due to an unexpected problem or difficulty at the time of birth)
- Caesarean - planned (booked ahead of time)

9.7
What was [baby number] 's date of birth?
Enter date

9. BABIES BORN SINCE FEBRUARY 2020 *Shown to all who have had ≥1 livebirth since February 2020*

9.8
Where did you give birth to [baby number]?
- Hospital (in an obstetric or midwifery unit)
- Birth centre or midwifery unit away from hospital
- At home
- On the way to hospital
- Somewhere else

9.9
Did you experience labour with [baby number]? (By labour, we mean a series of regular contractions of the uterus that the cervix open and thin)
- Yes, it started spontaneously
- Yes, but only after induction of labour (with or without membrane sweeps)
- No

9.10
What sex was [baby number]?
- Female
- Male
- Indeterminate
- Prefer not to answer

9.11
Was [baby number] born with any serious health problems likely to cause physical or mental problems in the long term?
- No → 9.13
- Yes → 9.12

9.12 *Shown if answer to 9.11 is Yes*
Please describe [baby number] 's health problem(s) for us.
Free text

9.13
How much did [baby number] weigh at birth? Please enter the weight in either grams (g) or pounds and ounces (lbs/oz)
- Weight in grams (g) → 9.14
- Weight in pounds and ounces (lb/oz) (g) → 9.15

9.14 *Shown if answer to 9.13 is weight in grams*
Please enter [baby number] weight in grams (g)
Free text
→ 9.16

9.15 *Shown if answer to 9.13 is weight in pounds and ounces*
Please enter [baby number] weight in pounds (lb) and ounces (oz)
Pounds (lb): Free text
Ounces (oz) : Free Text

9.16
Did [baby number] require care in a newborn care unit that separated him/her from you for four hours or more after birth?
- No
- Yes

9.17
Did [baby number] go home after birth, even if he/she had to stay in hospital for a period of time?
- No
- Yes

11. OPTIONAL SECTION 1: YOUR HEALTH AFTER BIRTH

Shown to all who agree to continue

11. OPTIONAL SECTION 1: YOUR HEALTH AFTER BIRTH

We would now like to ask some questions about your health, birth, and parenting during the COVID-19 pandemic and beyond.

- ○ I am happy to continue to share my views → 11.2
- ○ I wish to finish the questionnaire now → End

The following questions are about your health after giving birth since February 2020.
These questions are about your <u>Nth</u> pregnancy since February 2020 *(questions repeat for each pregnancy)*

11.3
Did you have community midwife visit you at home before your baby or babies were six weeks of age?
- ○ No
- ○ Yes

11.4
Since giving birth, until the time when your baby or babies were six weeks of age, did you have to return to your general practitioner, walk-in health centre, or hospital *urgently*? Please do not include your routine 6 week check after birth.
- ○ No
- ○ Yes

11.5
Since being at home after giving birth, until the time when your baby or babies were six weeks of age, have you had to stay in hospital for at least one night with a health problem?
- ○ No
- ○ Yes

11.6
These questions are still about your <u>Nth</u> pregnancy since February 2020.

Did you have stitches after giving birth? These could have been a result of a Caesarean delivery, episiotomy, or vaginal tear.
- ○ No → 11.10
- ○ Yes

11.7
Did you have an infection of your stitches after giving birth?
- ○ No → 11.10
- ○ Yes

11.8
Were you prescribed antibiotics for this infection?
- ○ No → 11.10
- ○ Yes

11.9
Did the midwife or doctor open up your wound?
- ○ No
- ○ Yes

11.10
Did you have a six-week check with your GP practice after giving birth?
- ○ No, I was not offered a six-week check
- ○ No, I was not able to book a six-week check
- ○ No, I did not want a six-week check (although I was offered the opportunity)
- ○ No, for another reason
- ○ Yes → 11.11
- ○ I can't remember

→ 11.13 unless otherwise specified

11.11 *Shown if 11.10 response is Yes*
How was your six-week check after the birth of [baby number] conducted with your GP practice?
- ○ In person

11. OPTIONAL SECTION 1: YOUR HEALTH AFTER BIRTH

- ○ By telephone
- ○ By video call
- ○ I can't remember

11.12 *Shown if 11.10 response is Yes*
How satisfied were you with the care you received at your six-week check after birth?
- ○ Very dissatisfied
- ○ Dissatisfied
- ○ Neither satisfied nor dissatisfied
- ○ Satisfied
- ○ Very satisfied

11.13
Did you breastfeed your baby or give expressed breastmilk (exclusively or in combination with formula) at any stage during the following time periods? *Choose the ONE best option*
- ○ Yes, up to the first 6 weeks
- ○ Yes, up to the first 3 months
- ○ Yes, up to the first 6 months
- ○ Yes, for 6 to 12 months
- ○ Yes, for more than 12 months and I am still breastfeeding
- ○ No → 11.14

→ 11.16 unless otherwise stated

11.14 *Shown if answer to 11.13 is No*
What were your reasons? *Choose ALL that apply.*
- ☐ I did not want to breastfeed
- ☐ I tried but was not able to breastfeed
- ☐ I didn't like breastfeeding when I breastfed my previous baby/babies
- ☐ I needed more support to breastfeed
- ☐ Other → 11.15

→ 11.16 unless otherwise stated

11.15 *Shown if answer to 11.14 is Other*
What were your reasons?
Free text

11.16
Was your decision not to breastfeed your baby (or give expressed breastmilk) or how long you did this influenced by the COVID-19 pandemic?
- ○ No
- ○ Yes
- ○ Unsure

11.17 *Shown if answer to 11.13 is Yes*
What were your reasons? *Choose ALL that apply.*
- ☐ It was easier to breastfeed (or give expressed breastmilk) when I was at home most of the time.
- ☐ I had more support to breastfeed (or give expressed breastmilk) from my partner.
- ☐ I was vaccinated and wanted to protect my baby from COVID-19 by giving him/her breastmilk.
- ☐ Other → 11.18
- ☐ Unsure

→ To 11.19 if not otherwise stated

11.18 *Shown if answer to 11.17 is Other*
Please tell us your reasons
Free text

13. OPTIONAL SECTION 2: BABY'S HEALTH AND PARENTING (BABY LOOP)

These questions are about [baby number] born since February 2020.

13.2

13. OPTIONAL SECTION 2: BABY'S HEALTH AND PARENTING (BABY LOOP)

Since coming home after birth, how has [baby number] been? *Choose ALL that apply.*

- ☐ There have been no serious problems → 13.4
- ☐ My baby has required many unscheduled visits to the GP → 13.4
- ☐ My baby has needed at least one admission to hospital → 13.4
- ☐ Sadly, my baby has passed away → 13.3

13.4
In the first six weeks of life, did you have concerns about your newborn [baby number] 's weight gain?

- o No → 13.6
- o Yes, some concerns →13.5
- o Yes, major concerns → 13.6

13.5 Shown if answer to 13.4 is 'yes, some concerns'
Were you able to get [baby number] weighed either by the community midwife, health visitor or at your GP surgery?

- o No
- o Yes, with difficulty
- o Yes, with ease

13.6
In the first six weeks of life, did you have concerns that [baby number] had jaundice?

- o No → 13.8
- o Yes

13.7 Shown if answer to 13.6 is yes
Were you able to have [baby number] looked at either by the community midwife, health visitor or at your GP surgery?

- o No
- o Yes

13.8
Did [baby number] have their six-week check at your GP?

- o No, I did not want a six-week check for my baby (although I was offered one)
- o No, I was not offered a six-week check for my baby
- o No, I was not able to book a six-week check for my baby
- o No, for another reason
- o No, the six-week check for my baby was combined with the two-month routine vaccination
- o Yes, face-to-face
- o Yes, by telephone or video appointment

13.9
Do you think that [baby number] has had COVID-19 since birth?

- o No
- o Yes

13.10
Will [baby number] attend childcare outside your home when available?
Choose the ONE best option.

- No, I have always planned to stay at home with my baby
- No, I do not want my baby to attend childcare in case he/she gets COVID-19
- No, I have family members who can care for my baby
- Yes
- Yes, my baby already attends childcare outside my home
- Yes, as my baby's grandparents are no longer able to look after him/her

13.11
How easy was it for you to book an appointment at your GP surgery for [baby number] 's routine (usual) vaccinations at 2, 3, 4, and 12 months of age (as relevant)?
Choose the ONE best option.

- o Very easy

13. OPTIONAL SECTION 2: BABY'S HEALTH AND PARENTING (BABY LOOP)

- ○ Easy
- ○ Neither easy nor difficult
- ○ Difficult
- ○ My baby's vaccinations were delayed as we were not able to attend the GP

13.12
Have you needed to contact your GP for an urgent same-day appointment, or needed to call NHS 111, because [baby number] was unwell during the first year of life?

- ○ No
- ○ Yes

13.13
What sort of appointment(s) did [baby number] have?
Choose ALL that apply.

- ☐ In-person → 13.14
- ☐ Video call → 13.15
- ☐ Telephone → 13.16

13.14
Please tell us what was positive about your in experience with the <u>in-person</u> appointment(s) [baby number] had, and what could have been done better?

Positive aspects: Free text
Could have been done better: Free text

→ 13.17

13.15
Please tell us what was positive about your in experience with the <u>video</u> appointment(s) [baby number] had, and what could have been done better?

Positive aspects: Free text
Could have been done better: Free text

→ 13.17

13.16
Please tell us what was positive about your in experience with the <u>telephone</u> appointment(s) [baby number] had, and what could have been done better?

Positive aspects: Free text
Could have been done better: Free text

→ 13.17

13.17
After the pandemic, how would you prefer to be seen for care of your child?

- ○ In-person
- ○ Video
- ○ Telephone

13.19
During the first year of life, have you needed to take [baby number] to Accident & Emergency (A&E), a walk-in centre, or an urgent care centre?

- ○ No → 13.22
- ○ Yes

13.20 *Shown if answer to 13.19 is Yes*
Why did you choose to take your baby for care at A&E, a walk-in centre, or an urgent care centre-?

- ○ My GP advised me to do so
- ○ NHS' 111' advised me to do so
- ○ I could not get an appointment with my GP
- ○ My baby was very sick
- ○ I wanted my baby to be seen in person and not by video appointment
- ○ Prefer not to say

13. OPTIONAL SECTION 2: BABY'S HEALTH AND PARENTING (BABY LOOP)

- Other → 13.21

→ 13.22 unless otherwise stated

13.21 *Shown if answer to 13.20 is Other*
Please explain your other reason
Free text

13.22
How many other children (older or younger) are there who live at home with you and your baby? Please include children who live with you part-time.
Enter number

The next questions are about your thoughts and feelings about your baby. Please tick one box only in answer to each question. There are 19 questions that should take about 10 minutes.

13.24
<u>When I am</u> caring for the baby, I get feelings of annoyance or irritation:
- Very frequently
- Frequently
- Occasionally
- Very rarely
- Never

13.25
<u>When I am</u> caring for the baby I get feelings that the child is deliberately being difficult or trying to upset me:
- Very frequently
- Frequently
- Occasionally
- Very rarely
- Never

13.26
<u>Over the last two weeks</u> I would describe my feelings for the baby as:
- Dislike
- No strong feeling towards the baby
- Slight affection
- Moderate affection
- Intense affection

13.27
<u>Regarding my overall</u> level of interaction with the baby I:
- Feel very guilty that I am not more involved
- Feel moderately guilty that I am not more involved
- Feel slightly guilty that I am not more involved
- I don't have any guilty feelings regarding this

13.28
When I interact with the baby I feel:
- Very incompetent and lacking in confidence
- Moderately incompetent and lacking in confidence
- Moderately competent and confident
- Very competent and confident

13.29
<u>When I am</u> with the baby I feel tense and anxious:
- Very frequently
- Frequently
- Occasionally
- Almost never

13.30

13. OPTIONAL SECTION 2: BABY'S HEALTH AND PARENTING (BABY LOOP)

When I am with the baby and other people are present, I feel proud of the baby:
- Very frequently
- Frequently
- Occasionally
- Almost never

13.31
I try to involve myself as much as I possibly can PLAYING with the baby:
- This is true
- This is untrue

13.32
When I have to leave the baby:
- I usually feel rather sad (or it's difficult to leave)
- I often feel rather sad (or it's difficult to leave)
- I have mixed feelings of both sadness and relief
- I often feel rather relieved (and it's easy to leave)
- I usually feel rather relieved (and it's easy to leave)

13.33
When I am with the baby:
- I always get a lot of enjoyment/satisfaction
- I frequently get a lot of enjoyment/satisfaction
- I occasionally get a lot of enjoyment/satisfaction
- I very rarely get a lot of enjoyment/satisfaction

13.34
When I am not with the baby, I find myself thinking about the baby:
- Almost all the time
- Very frequently
- Frequently
- Occasionally
- Not at all

13.35
When I am with the baby:
- I usually try to prolong the time I spend with him/her
- I usually try to shorten the time I spend with him/her

13.36
When I have been away from the baby for a while and I am about to be with him/her again, I usually feel:
- Intense pleasure at the idea
- Moderate pleasure at the idea
- Mild pleasure at the idea
- No feelings at all about the idea
- Negative feelings about the idea

13.37
I now think of the baby as:
- Very much my own baby
- A bit like my own baby
- Not yet really my own baby

13.38
Regarding the things that we have had to give up because of the baby:
- I find that I resent it quite a lot
- I find that I resent it a moderate amount
- I find that I resent it a bit
- I don't resent it at all

13.39

13. OPTIONAL SECTION 2: BABY'S HEALTH AND PARENTING (BABY LOOP)

<u>Over the past</u> three months, I have felt that I do not have enough time for myself or to pursue my own interests:

- Almost all the time
- Very frequently
- Occasionally
- Not at all

13.40
Taking care of this baby is a heavy burden of responsibility. I believe this is:

- Very much so
- Somewhat so
- Slightly so
- Not at all

13.41
<u>I trust my own judgement</u> in deciding what the baby needs:

- Almost never
- Occasionally
- Most of the time
- Almost all of the time

13.42
<u>Usually when I</u> am with the baby:

- I am very impatient
- I am a bit impatient
- I am moderately patient
- I am extremely patient

13.43
Do you think that your baby's development has been affected by lockdown? Choose one best option.

- No, not at all
- No, not much
- Yes, for the better
- Yes, for the worse
- Yes, for the better in some ways and for the worse in other ways
- Don't really know

END OF BASELINE QUESTIONNAIRE

Shown to all

Is there anything else that you would like to share with us related to planning pregnancy, pregnancy, and the time after birth since February 2020 when the COVID-19 pandemic began in the UK?

Free text

→ End of survey

FOLLOW UP QUESTIONNAIRE

- Blocks 2 to 8 were shown to all respondents.
- Blocks 9 was shown to respondents who reported one or more livebirths since February 2020.
- Blocks 11 and 13 were optional sections shown to respondents who reported one or more livebirths since February 2020 and who agreed to continue.
- The questionnaire was administered using REDCap (Research Electronic Data Capture) hosted at King's College London.

2. PREGNANCY STATUS

These first questions are about pregnancy and contemplating pregnancy.
Questions about pregnancy and pregnancy planning can sometimes be sensitive. If you find any of the questions upsetting, please accept our apologies. We ask these questions because they are important in helping us to understand how the pandemic has affected women of childbearing age.

2.1
Which of the following best describes your current pregnancy status?
- ○ I have never been pregnant
- ○ I am not pregnant now, but I have been in the past (including miscarriage, ectopic pregnancy, termination, stillbirths, and livebirths)
- ○ I am pregnant now → 2.6

2.2
Are you thinking about becoming pregnant?
- ○ No → block 3 if never pregnant; 2.7 if pregnant in past
- ○ Yes

2.3
Are you trying to become pregnant now?
- ○ No → 2.7
- ○ Yes

2.4
Are you taking folic acid for pregnancy planning?
- ○ No
- ○ Yes

2.5
For how many months have you been trying?
Free text (integer)

2.6
What is your due date?
Enter date

We would now like to ask you about your pregnancy history. *Shown if pregnant now or pregnant in the past*

2.7
How many times have you ever been pregnant? Please include your current pregnancy if you are pregnant today. Enter your answer as a number e.g., 1 for once, 2 for twice etc.
Free text

Have you had a pregnancy that has ended during the following time periods?
Please enter the number of any previous miscarriages, elective terminations, ectopic pregnancies (a tubal pregnancy), stillborn, or liveborn babies you have had.

2.8
Miscarriages

Up to and including 31-Jan-2020	
Between 1-Feb-2020 and 30-Sep-2021	
Since 1-Oct-2021	

2. PREGNANCY STATUS

Total	*Sums automatically*

2.9
Elective terminations

Up to and including 31-Jan-2020	
Between 1-Feb-2020 and 30-Sep-2021	
Since 1-Oct-2021	
Total	*Sums automatically*

2.10
Ectopic (tubal) pregnancies

Up to and including 31-Jan-2020	
Between 1-Feb-2020 and 30-Sep-2021	
Since 1-Oct-2021	
Total	*Sums automatically*

2.11
Stillborn babies

Up to and including 31-Jan-2020	
Between 1-Feb-2020 and 30-Sep-2021	
Since 1-Oct-2021	
Total	*Sums automatically*

2.12
Liveborn babies

Up to and including 31-Jan-2020	
Between 1-Feb-2020 and 30-Sep-2021	
Since 1-Oct-2021	
Total	*Sums automatically*

Maternity leave

2.13
Shows if currently pregnant and due date within next 2 months or had liveborn or stillborn since Oct 21
Are you currently on maternity leave?
- Yes
- No

2.14
Shows if had liveborn or stillborn baby since Feb 2020
Were you on maternity leave on [baseline date] (the last time you completed the questionnaire)?
- Yes
- No

3. COVID VACCINATIONS

We'd now like to ask you about any COVID-19 vaccine injections you may have received.

3.1
How many doses of a COVID-19 vaccine have you had?
Please enter the number of COVID-19 vaccinations/boosters in the box below. If you have only had a vaccine as part of a clinical trial, do not include any placebo (dummy vaccine) doses.

3. COVID VACCINATIONS

Enter number
→ 3.4 if ≥ 1 vaccine on record
→ 3.5 if no vaccine data on record

3.4 *For each additional dose*
If vaccine data on record
VACCINE N: You told us previously that your Nth vaccination was on [piped vaccine date]. Is this correct?
- 1, Yes - this is correct → Next vaccine or block
- 0, No - I need to correct the date

3.5 *For each additional dose (up to 6)*
What was the date of your Nth COVID-19 vaccination?
Enter date

4. VIEWS ON COVID-19 VACCINATION

In this section, we would like to ask your views on vaccination.

4.1 *Shown if 3.1 = 0*
Please tell us why you have not had, or would not have, a COVID-19 vaccination. *Choose ALL that apply.*
- ☐ I have a health condition that means that I am not able to have a vaccination/booster
- ☐ I have not been offered a vaccination/booster
- ☐ I took part in a vaccine trial
- ☐ I've had COVID and think I am now protected by antibodies (have immunity)
- ☐ Religious reasons
- ☐ Personal belief/philosophical reasons
- ☐ My midwife has advised against it
- ☐ My doctor has advised against it
- ☐ I am not able to have vaccinations
- ☐ Planning pregnancy
- ☐ Receiving fertility treatment
- ☐ Concerned it may affect my me and my baby
- ☐ Concerned that it may not be safe during breastfeeding
- ☐ Concerned about the long-term side effects
- ☐ Do not know enough about it
- ☐ Concerned about the number of vaccine boosters I would need
- ☐ Illness/medication
- ☐ Do not think it will be available to me
- ☐ I do not think that I am at sufficient risk
- ☐ I'm concerned about the vaccine development and approvals process
- ☐ I feel that the possible risks are unacceptable.
- ☐ Natural (infection) is better
- ☐ I'm not concerned about getting seriously ill
- ☐ I don't feel the evidence of benefit is reliable
- ☐ Do not think it will work
- ☐ Concerned about adverse reaction
- ☐ I am against all vaccination → 4.2
- ☐ Other reason → 4.3
- ☐ Prefer not to answer

4.2
Please share your views with us about why you are against all vaccination.
Free text

4.3
Please share the other reason(s) why you did not have a COVID-19 vaccination.
Free text

4.4 *Shown if 3.1 ≥ 1*

4. VIEWS ON COVID-19 VACCINATION

Why did you have a COVID-19 vaccine? *Choose ALL that apply.*

- ☐ I am worried about getting COVID-19
- ☐ I am worried about getting really sick from COVID-19
- ☐ I have had COVID-19 and I don't want to get it again
- ☐ I have had a family/friend who was very sick or who died from COVID-19
- ☐ I am worried about spreading COVID-19 to others, including family and friends
- ☐ I think that the benefits outweigh the risks
- ☐ There is benefit if most people are vaccinated
- ☐ The risks to me of vaccination are very small
- ☐ I support vaccination when it is available
- ☐ I have an Illness or take medication that makes me more vulnerable to COVID-19
- ☐ I am pregnant and want to keep me and my baby safe
- ☐ I think that it is my responsibility as a member of my community
- ☐ I want to be active in my community again, without fear of getting sick from COVID-19
- ☐ I received an invitation from the NHS/my GP
- ☐ To travel abroad
- ☐ For my job
- ☐ The government recommends it
- ☐ Other reason → 4.5
- ☐ Prefer not to answer

4.5

Please share the other reason(s) why you had a COVID-19 vaccine.

Free text

Future COVID-19 boosters

4.6

If you were to be offered a COVID-19 booster vaccination in the future, would you have one?

- ○ Yes → 4.9
- ○ No → 4.7
- ○ Unsure → 4.7

4.7

Please tell us why you would not have or are unsure about having a COVID-19 booster vaccination in the future. *Choose ALL that apply.*

- ☐ I have a health condition that means that I am not able to have a vaccination/booster
- ☐ I have not been offered a vaccination/booster
- ☐ I took part in a vaccine trial
- ☐ I've had COVID and think I am now protected by antibodies (have immunity)
- ☐ Religious reasons
- ☐ Personal belief/philosophical reasons
- ☐ My midwife has advised against it
- ☐ My doctor has advised against it
- ☐ I am not able to have vaccinations
- ☐ Planning pregnancy
- ☐ Receiving fertility treatment
- ☐ Concerned it may affect my me and my baby
- ☐ Concerned that it may not be safe during breastfeeding
- ☐ Concerned about the long-term side effects
- ☐ Do not know enough about it
- ☐ Concerned about the number of vaccine boosters I would need
- ☐ Illness/medication
- ☐ Do not think it will be available to me
- ☐ I do not think that I am at sufficient risk
- ☐ I'm concerned about the vaccine development and approvals process
- ☐ I feel that the possible risks are unacceptable.

4. VIEWS ON COVID-19 VACCINATION

- ☐ Natural (infection) is better
- ☐ I'm not concerned about getting seriously ill
- ☐ I don't feel the evidence of benefit is reliable
- ☐ Do not think it will work
- ☐ Concerned about adverse reaction
- ☐ I am against all vaccination
- ☐ Other reason → 4.8
- ☐ Prefer not to answer

4.8
Please share the other reason(s) why you would not have or are unsure about having a COVID-19 booster vaccination in the future.
Free text

4.9
Why would you have a COVID-19 booster vaccine in the future? *Choose ALL that apply.*

- ☐ I am worried about getting COVID-19
- ☐ I am worried about getting really sick from COVID-19
- ☐ I have had COVID-19 and I don't want to get it again
- ☐ I have had a family/friend who was very sick or who died from COVID-19
- ☐ I am worried about spreading COVID-19 to others, including family and friends
- ☐ I think that the benefits outweigh the risks
- ☐ There is benefit if most people are vaccinated
- ☐ The risks to me of vaccination are very small
- ☐ I support vaccination when it is available
- ☐ I have an Illness or take medication that makes me more vulnerable to COVID-19
- ☐ I am pregnant and want to keep me and my baby safe
- ☐ I think that it is my responsibility as a member of my community
- ☐ I want to be active in my community again, without fear of getting sick from COVID-19
- ☐ I received an invitation from the NHS/my GP
- ☐ To travel abroad
- ☐ For my job
- ☐ The government recommends it
- ☐ Other reason → 4.10
- ☐ Prefer not to answer

4.10
Please share the other reason(s) why you would have a COVID-19 booster vaccine.
Free text

5. OTHER VACCINATIONS

These questions are about other types of vaccination.

5.1
Did you have a flu (influenza) vaccination last autumn/winter (i.e., since 1st September 2021)?

- ○ No → 5.3
- ○ Yes → 5.2
- ○ Not sure → 5.3/next block

5.2
What date did you have your flu vaccine? If you do not remember exactly, please put your best estimate.
Free text

5.3 *Shown if pregnant now or had ≥1 liveborn since Oct 2021*
In your current pregnancy (or most recent pregnancy), have you had, or do you plan to have, a whooping cough (pertussis) vaccination?

- ○ Yes
- ○ No

- Not sure

6. COVID-19 HISTORY

These questions are about having COVID-19.

6.1
Have you had COVID-19 since [*baseline survey date*]?
- Yes
- No → block 7

6.2
How many times have you had a COVID-19 infection since [*baseline survey date*]?
Drop down list

6.3 *Repeats for number of infections shown in 6.2*
Please enter the date when you got (or might have got) your Nth COVID-19. If you do not remember exactly, please put your best estimate.
Enter date

6.4 *Repeats for number of infections shown in 6.2*
How was your Nth infection of COVID-19 confirmed?
- A positive test
- Medical advice
- Strong personal suspicion
- Unsure
- Prefer not to say → next episode or section

6.5 *Repeats for number of infections shown in 6.2*
At the time of having or possibly having COVID-19 for the Nth time, did you have symptoms of COVID-19, such as fever, cough, loss of taste or smell, or sneezing?
- No → next repeat block or block 7
- Yes

6.6 *Repeats for number of infections shown in 6.2*
How long have you had/did you have COVID-19 symptoms overall following your Nth infection? Please include time spent with mild symptoms and the time in between symptoms if these came and went or are still ongoing.
- I did not have any symptoms
- Less than 2 weeks
- 2-4 weeks
- 4-12 weeks
- 3-6 months
- 6-12 months
- 12-18 months
- 18-24 months
- More than 24 months

6.7 *Repeats for number of infections shown in 6.2*
Have you ever had to stay in hospital because of COVID-19 symptoms when you had COVID-19 for the Nth time?
- Yes
- No
- Prefer not to answer

7. MENTAL HEALTH

These next questions are about how you are feeling.

7.1
Please read each item and select the reply which comes closest to how you have been feeling. Don't take too long over your replies. Your immediate reaction to each item will probably be more accurate than a long thought-out response.
Over the last 2 weeks, HOW OFTEN have you been bothered by the following problems:

7. MENTAL HEALTH

	Not at all	Several days	More than half the days	Nearly every day
Little interest or pleasure in doing things e_pg_fu1_phq2a				
Feeling down, depressed or hopeless e_pg_fu1_phq2b				
Feeling nervous, anxious or on edge e_pg_fu1_gad2a				
Not being able to stop or control worrying e_pg_fu1_gad2b				

9. BABIES BORN SINCE OCTOBER 2021

The next section is about your liveborn baby or babies born since October 2021.

9.1
Earlier, you told us earlier that you have had [piped in number] liveborn baby or babies since October 2021. To make sure we show you the right questions, please let us know if this number is correct.

- Yes, this is correct → 9.3
- No, I need to correct this

9.2
How many liveborn babies have you had since October 2021?

Enter number

The next questions are about your Nth liveborn baby since October 2021.
In these next questions, your baby will be referred to as baby N.

9.3 *Repeats for number of liveborn shown in 9.1*
Was baby N born from a single or multiple baby pregnancy?

- Single – only ONE baby
- Multiple – one of TWO babies (twins)
- Multiple – one of THREE babies (triplets)
- Multiple – one of FOUR babies (quadruplets)

9.4 *Repeats for number of liveborn shown in 9.1*
How was baby N born?

- Vaginal birth with assistance (such as, forceps, vacuum, or breech delivery)
- Vaginal birth with no assistance (including physiological breech birth)
- Caesarean birth - emergency (due to an unexpected problem or difficulty at the time of birth)
- Caesarean birth - planned (booked ahead of time)

9.5 *Repeats for number of liveborn shown in 9.1*
What was baby N's date of birth?

Enter date

9.6 *Repeats for number of liveborn shown in 9.1*
Where did you give birth to baby N?

- Hospital labour ward or theatre
- Hospital-based in an obstetric or midwifery unit) or birth centre
- Birth centre or midwifery unit away from hospital with facilities to offer caesarean birth
- At home
- On the way to hospital
- Somewhere else

9.7 *Repeats for number of liveborn shown in 9.1*
Did you experience labour with baby N? (By labour, we mean a series of regular contractions of the uterus that the cervix open and thin)

- Yes, it started spontaneously
- Yes, but only after induction of labour (with or without membrane sweeps)
- No

9. BABIES BORN SINCE OCTOBER 2021

9.8 *Repeats for number of liveborn shown in 9.1*
What sex was baby N?
- ○ Female
- ○ Male
- ○ Indeterminate
- ○ Prefer not to answer

9.9 *Repeats for number of liveborn shown in 9.1*
How much did baby N weigh at birth? Please choose the closest weight from the list.
List

9.10 *Repeats for number of liveborn shown in 9.1*
Do you think that baby N has had COVID-19 since birth?
- ○ Yes
- ○ No

The next questions ask about health problems after birth and neonatal death (deaths soon after birth)

9.11 *Repeats for number of liveborn shown in 9.1*
Was baby N born with any serious health problems likely to cause physical or mental problems in the long term?
- ○ No → 9.12
- ○ Yes

9.12 *Repeats for number of liveborn shown in 9.1*
Please describe baby N's health problem(s) for us.
Free text

9.13 *Repeats for number of liveborn shown in 9.1*
Did baby N require care in a newborn care unit that separated him/her from you for four hours or more after birth?
- ○ No
- ○ Yes

9.14 *Repeats for number of liveborn shown in 9.1*
Did baby N go home after birth, even if he/she had to stay in hospital for a period of time?
- ○ Yes - my baby was well enough to come home with me
- ○ No - sadly my baby died in hospital before coming home

9.15 *Shown if baby was able to go home*
Since coming home after birth, how has baby 1 been? *Choose ALL that apply.*
- ☐ There have been no serious problems
- ☐ My baby has required many unscheduled visits to the GP
- ☐ My baby has needed at least one admission to the hospital
- ☐ Sadly, my baby has passed away since coming home

10. YOUR HEALTH AFTER BIRTH

The following questions are about your health after giving birth since October 2021.

10.1
Did you have any problems with the following during your pregnancy?
Please choose all options that are relevant and choose 'no' if you had none of the conditions listed.
- ☐ High blood pressure
- ☐ Gestational diabetes → 10.3
- ☐ Urine infection
- ☐ Preterm birth
- ☐ Other → 10.2
- ☐ No

→ 10.3, unless otherwise stated

10.2
Please let us know about the other health problem(s) you had during your pregnancy
Free text

10. YOUR HEALTH AFTER BIRTH

10.3
Did you have a blood sample taken about six weeks after birth to test for high blood sugar?
- No
- Yes
- Don't know
- Not yet enough time has passed since the birth, but I plan to

10.4
Did you have community midwife, maternity support worker or health visitor visit you at home before your baby or babies were six weeks of age?
- No
- Yes

10.5
Since giving birth, until the time when your baby or babies were six weeks of age, did you have to return to your general practitioner, walk-in health centre, or hospital *urgently*? Please do not include your routine 6 week check after birth.
- No
- Yes

10.6
Since being at home after giving birth, until the time when your baby or babies were six weeks of age, have you had to stay in hospital for at least one night with a health problem?
- No
- Yes

10.7
Did you have stitches after giving birth? These could have been a result of a Caesarean delivery, episiotomy, or vaginal tear.
- No
- Yes

10.8
Did you have an infection of your stitches after giving birth?
- No
- Yes

10.9
Were you prescribed antibiotics for this infection?
- No → 11.10
- Yes

10.10
Did the midwife or doctor open up your wound?
- No
- Yes

10.11
Did you have a six-week check with your GP practice after giving birth?
- No, I was not offered a six-week check → 10.14
- No, I was not able to book a six-week check → 10.14
- No, I did not want a six-week check (although I was offered the opportunity) → 10.14
- No, it has not yet been six weeks since giving birth → 10.14
- No, for another reason → 10.14
- Yes
- I can't remember → 10.14

10.12
How was your six-week check after the birth conducted with your GP practice?
- In person
- By telephone

10. YOUR HEALTH AFTER BIRTH

- ○ By video call
- ○ I can't remember

10.13
How satisfied were you with the care you received at your six-week check after birth?
- ○ Very dissatisfied
- ○ Dissatisfied
- ○ Neither satisfied nor dissatisfied
- ○ Satisfied
- ○ Very satisfied

These questions are about breastfeeding.

10.14
Did you breastfeed your baby or give expressed breastmilk (exclusively or in combination with formula) at any stage during the following time periods? *Choose the ONE option that fits your experience most closely*
- ○ Yes, up to the first 6 weeks
- ○ Yes, up to the first 3 months
- ○ Yes, up to the first 6 months
- ○ Yes, for 6 to 12 months
- ○ Yes, for more than 12 months or I am still breastfeeding
- ○ No

10.15
What were your reasons for not breastfeeding? *Choose ALL that apply.*
- ☐ I did not want to breastfeed
- ☐ I tried but was not able to breastfeed
- ☐ I didn't like breastfeeding when I breastfed my previous baby/babies
- ☐ I needed more support to breastfeed
- ☐ Other

10.16
What were your other reasons?
Free text

10.17
Was your decision not to breastfeed your baby (or give expressed breastmilk) or how long you did this influenced by the COVID-19 pandemic?
- ○ No
- ○ Yes
- ○ Unsure

10.18
What were your reasons for breastfeeding? *Choose ALL that apply.*
- ○ It was easier to breastfeed (or give expressed breastmilk) when I was at home most of the time.
- ○ I had more support to breastfeed (or give expressed breastmilk) from my partner.
- ○ I was vaccinated and wanted to protect my baby from COVID-19 by giving him/her breastmilk.
- ○ Other
- ○ Unsure

10.19
Please tell us your other reasons
Free text

10.20
Is there anything else that you would like to share with us related to the pandemic, vaccination, planning pregnancy, pregnancy, or the time after birth since 01-10-2021?
Free text

-END-

Table 1: Alterations of Baseline Survey for Follow-up Survey

Section	Row	Question	Baseline Question ID	Baseline notes	Follow-up Question ID	Follow-up notes
**Pregnancy Status & History**	1	Which of the following best describes your current pregnancy status?	2.2		2.1	
	2	Are you thinking about becoming pregnant?	2.3		2.2	
	3	Are you trying to become pregnant now?	2.4		2.3	
	4	Are you taking folic acid for pregnancy planning?	X		2.4	
	5	For how many months have you been trying?	2.5		2.5	
	6	Have you had treatment from a doctor to help you become pregnant?	2.6		X	
	7	Have your pregnancy plans changed at all because of the pandemic?	2.7		X	
	8	What is your due date?	2.8		2.6	
	9	How many times have you ever been pregnant? Please include the current pregnancy if you are pregnant today, as well as any previous miscarriages, elective terminations, ectopic pregnancies, stillborns, and liveborns.	2.9		X	
	10	We would now like to ask you about your pregnancies.<br>Have you ever had any of the following? Please enter the number of times below:<br>-Miscarriage<br>-Elective termination<br>-Ectopic (tubal) pregnancy<br>-Stillborn baby<br>-Liveborn baby<br>-Total (calculated automatically)	2.10	Sands website shown for stillbirths	X	
	11	How many times have you been pregnant?<br>Please include your current pregnancy if you are pregnant today, as well as any previous miscarriages, elective terminations, ectopic pregnancies (a tubal pregnancy), stillborns, and liveborns you have had.<br>Enter the numbers in the boxes below.<br>Please note the different time periods (Ever - Since Feb 2020 - Since Oct 2021)	X		2.7	Reformatted baseline question 2.10 Sands website

Section	Row	Question	Baseline Question ID	Baseline notes	Follow-up Question ID	Follow-up notes
		-Miscarriage<br>-Elective termination<br>-Ectopic (tubal) pregnancy<br>-Stillborn baby<br>-Liveborn baby<br>-Totals (calculated automatically)				shown for stillbirths Miscarriage Association website shown for miscarriages or ectopics
	12	How many times have you been pregnant since February 2020 (when the pandemic started in the UK)?	2.12		X	
	13	How many liveborn babies have you had since February 2020 ?<br>Enter the number in the box below.	2.13		X	
**COVID vaccinations**	14	Have you had a COVID-19 vaccination?	3.2		X	
	15	The last time you completed the questionnaire (on [BASELINE_SURVEY_DATE]) you told us you had received [NUMBER_BASELINE_COVID_VACCINATIONS]. Have you since had any more COVID-19 vaccination doses?	X		3.1	Displays N doses reported at baseline
	16	How many COVID-19 vaccine doses have you had?	3.3		Add	Additional doses since baseline
	17	How many doses of a COVID-19 vaccine have you had since [BASELINE_SURVEY_DATE]? Please enter the number of new doses in the box below. If you have only had a vaccine as part of a clinical trial, do not include any placebo (dummy vaccine) doses.	X		3.2	

Section	Row	Question	Baseline Question ID	Baseline notes	Follow-up Question ID	Follow-up notes
**COVID vaccination doses**	18	What is the name of the [nth] vaccine you received?	4.2		Add	
	19	What date did you have your [nth] [name of vaccine] injection? If you do not remember exactly, please put your best estimate.	4.3		3.3	Displays dates entered at baseline. Can add up to 5 dates at follow-up
	20	In the hours to days following your vaccination, did you experience any symptoms near the injection site after your [nth] injection [name of vaccine]?	4.4		X	
	21	Did you experience any other symptoms after your [nth] injection?	4.5		X	
	22	Was your headache like any of the following?	4.6		X	
	23	Did your feeling of being hot or shivery, or having a fever last longer than two days?	4.7		X	
	24	Did you seek medical attention for any of these symptoms?	4.8		X	
	25	Please tell us what you were told	4.9		X	
**Vaccination views**	26	Would you have a COVID-19 vaccine if offered?	5.2		X	
	27	Please tell us why you have not had or would not have a COVID-19 vaccination, if offered.	5.3		4.1	
	28	Please share your views with us about why you are against all vaccination.	5.4		4.2	
	29	Please specify the other reason(s) why you would not have had a COVID-19 vaccination	5.5		4.3	
	30	Why would or why did you have a COVID-19 vaccine?	5.6		4.4	

Section	Row	Question	Baseline Question ID	Baseline notes	Follow-up Question ID	Follow-up notes
	31	Please specify the other reason(s) why you would or did have a COVID-19 vaccination.	5.7		4.5	
	32	Do you think that pregnant or breastfeeding women should be recommended to receive the COVID-19 vaccine?	5.8		X	
**Other vaccination**	33	Have you had a flu (influenza) vaccination this autumn/ winter (i.e., since 1st September 2021)?	3b.1		X	
	34	Did you have a flu (influenza) vaccination last autumn/winter (i.e., since 1st September 2021)?	X		5.1	Had flu vaccination in Sep 21
	35	Would you have a flu vaccine if offered this autumn/ winter?	3b.2		X	
	36	What date did you have your flu vaccine. If you do not remember exactly, please put your best estimate.	3b.3		5.2	
	37	Have you had, or plan to have a whooping cough (pertussis) vaccination during your current or most recent pregnancy?	X		5.3	
**COVID history**	38	Do you think that you currently have or have ever had COVID-19?	6.2		6.3	Follow-up question wording: How was your Nth infection of COVID-19 confirmed?
	39	How many times have you had a COVID-19 infection? Please include infections confirmed by positive test, medical advice or strong personal suspicion.	X		6.1	
	40	Do you know the date when you first got (or might have got) COVID-19?	6.3		X	

Section	Row	Question	Baseline Question ID	Baseline notes	Follow-up Question ID	Follow-up notes
	41	Please enter the date when you first got (or might have got) COVID-19. If you do not remember exactly, please put your best estimate.	6.4		6.2	
	42	At the time of having or possibly having COVID-19, did you have symptoms of COVID-19, such as fever, cough, loss of taste or smell, or sneezing?	6.5		6.4	
	43	Have you ever had to stay in hospital because of COVID-19 symptoms?	6.6		6.5	
	44	Do you think you have had COVID-19 more than once?	6.7		X	
	45	Do you know the date when you got (or might have got) COVID-19 the second time?	6.8		X	
	46	Please enter the date when you got (or might have got) COVID-19 the second time. If you do not remember exactly, please put your best estimate.	6.9		X	
	47	How long have you had, or did you have COVID-19 symptoms overall? Please include time spent with mild symptoms and the time in between symptoms if these have been coming and going.	6.1		6.6	
**General and Mental Health**	48	In general, do you have any health problems that require you to stay at home?	7.2		X	
	49	Do you have any of the following health problems?	7.3		X	
	50	What type of diabetes do you have?	7.4		X	
	51	Have you ever been told that you have cancer?	7.5		X	
	52	What type(s) of cancer do you have now, or did you have in the past?	7.6		X	
	53	Are you using any of the following forms of contraception?	7.7		X	
	54	Do you regularly take immunosuppressant medications (including steroids, methotrexate, or biologics)?	7.8		X	
	55	Do you smoke?	7.9		X	
	56	Over the last 2 weeks, HOW OFTEN have you been bothered by the following problems	7.11	PHQ2/ GAD2 Samar	7.1	PHQ2/ GAD2 Samari

Section	Row	Question	Baseline Question ID	Baseline notes	Follow-up Question ID	Follow-up notes
				itans website and phone number shown for screen positive scores		tans website and phone number shown for screen positive scores
	57	Have you had any concerns about your mental health or emotional well-being since the beginning of the pandemic (in February 2020 in the UK)?	7.12		X	
	58	Have you ever been diagnosed with a mental health condition?	7.13		X	
	59	Please tell us which of the following you have been diagnosed with.	7.14		X	
**COVID-19 Risk Factors**	60	Are you a health care worker (including hospital, elderly care or in the community)?	8.2		X	
	61	Have you ever interacted (in person or face-to-face) with patients with confirmed or suspected COVID-19 infection?	8.3		X	
	62	Since the COVID-19 pandemic began, have you used personal protective equipment (PPE) at work?	8.4		X	
	63	Have you EVER been exposed to someone with confirmed or suspected COVID-19 infection (such as co-workers, family members, or others)?	8.5		X	
	64	Have you been self-isolating at all over the last week?	8.6		X	
	65	In the last week, did you wear a face mask (or other face covering) indoors when away from home (other than at work)?	8.7		X	
**Babies born since Feb 2020**	66	Earlier, you told us earlier that you have had [e_pg_fu1_numb_liveb_oct21] liveborn baby or babies since October 2021. To make sure we show you the right questions, please let us know if this number is correct.	X		9.1	

Section	Row	Question	Baseline Question ID	Baseline notes	Follow-up Question ID	Follow-up notes
	67	How many liveborn babies have you had since October 2021?	X		9.2	Allow correction before continuing
	68	Was [baby number] born from a single or multiple baby pregnancy?	9.3		X	
	69	How was [baby number] born?	9.6		9.4	
	70	What was [baby number]'s date of birth?	9.7		9.5	
	71	Where did you give birth to [baby number]?	9.8		9.6	
	72	Did you experience labour with [baby number]? (By labour, we mean a series of regular contractions of the uterus that the cervix open and thin)	9.9		9.7	
	73	What sex was [baby number]?	9.10		9.8	
	74	Was [baby number] born with any serious health problems likely to cause physical or mental problems in the long term?	9.11		9.9	
	75	Please describe [baby number]'s health problem(s) for us.	9.12		9.10	
	76	How much did [baby number] weigh at birth? Please enter the weight in either grams (g) or pounds and ounces (lbs/oz)	9.13		X	
	77	Please enter [baby number] weight in grams (g)	9.14		9.11	Using one list of weight in g and lb/oz for follow-up
	78	Please enter [baby number] weight in pounds (lb) and ounces (oz)	9.15		X	
	79	Did [baby number] require care in a newborn care unit that separated him/her from you for four hours or more after birth?	9.16		9.12	
	80	Did [baby number] go home after birth, even if he/she had to stay in hospital for a period of time?	9.17		9.13	

Section	Row	Question	Baseline Question ID	Baseline notes	Follow-up Question ID	Follow-up notes
	81	Since coming home after birth, how has [baby number] been?	13.2		9.14	Moved from section 13 (determines if baby has passed away) Sands message displayed if baby deceased
**Health after birth**	82	Did you have any problems with the following during your pregnancy with [baby number]?<br>Please choose all options that are relevant, and choose 'no' if you had none of the conditions listed.	9.4	Was in section 9	10.1	
	83	Please let us know about the other health problem(s) you had with [baby number].	9.5	Was in section 9	10.2	
	84	Did you have a blood sample taken about six weeks after birth to test for high blood sugar?	X		10.3	
	85	Did you have community midwife visit you at home before your baby or babies were six weeks of age?	11.3		10.4	
	86	Since giving birth, until the time when your baby or babies were six weeks of age, did you have to return to your general practitioner, walk-in health centre, or hospital urgently? Please do not include your routine 6 week check after birth.	11.4		10.5	
	87	Since being at home after giving birth, until the time when your baby or babies were six weeks of age, have you had to stay in hospital for at least one night with a health problem?	11.5		10.6	

Section	Row	Question	Baseline Question ID	Baseline notes	Follow-up Question ID	Follow-up notes
	88	Did you have stitches after giving birth? These could have been a result of a Caesarean delivery, episiotomy, or vaginal tear.	11.6		10.7	
	89	Did you have an infection of your stitches after giving birth?	11.7		10.8	
	90	Were you prescribed antibiotics for this infection?	11.8		10.9	
	91	Did the midwife or doctor open up your wound?	11.9		10.10	
	92	Did you have a six-week check with your GP practice after giving birth?	11.1		10.11	
	93	How was your six-week check after the birth of [baby number] conducted with your GP practice?	11.11		10.12	
	94	How satisfied were you with the care you received at your six-week check after birth?	11.12		10.13	
**Breastfeeding**	95	Did you breastfeed your baby or give expressed breastmilk (exclusively or in combination with formula) at any stage during the following time periods?	11.13		10.14	
	96	What were your reasons for not breastfeeding?	11.14		10.15	
	97	What were your other reasons?	11.15		10.16	
	98	Was your decision not to breastfeed your baby (or give expressed breastmilk) or how long you did this influenced by the COVID-19 pandemic?	11.16		10.17	
	99	What were your reasons for breastfeeding?	11.17		10.18	
	100	Please tell us your other reasons	11.18		10.19	
**Baby's health and parenting**	101	In the first six weeks of life, did you have concerns about your newborn [baby number]'s weight gain?	13.4		X	
	102	Were you able to get [baby number] weighed either by the community midwife, health visitor or at your GP surgery?	13.5		X	
	103	In the first six weeks of life, did you have concerns that [baby number] had jaundice?	13.6		X	

Section	Row	Question	Baseline Question ID	Baseline notes	Follow-up Question ID	Follow-up notes
	104	Were you able to have [baby number] looked at either by the community midwife, health visitor or at your GP surgery?	13.7		X	
	105	Did [baby number] have their six-week check at your GP?	13.8		X	
	106	Do you think that [baby number] has had COVID-19 since birth?	13.9		X	
	107	Will [baby number] attend childcare outside your home when available?	13.1		X	
	108	How easy was it for you to book an appointment at your GP surgery for [baby number]'s routine (usual) vaccinations at 2, 3, 4, and 12 months of age (as relevant)?	13.11		X	
	109	Have you needed to contact your GP for an urgent same-day appointment, or needed to call NHS 111, because [baby number] was unwell during the first year of life?	13.12		X	
	110	What sort of appointment(s) did [baby number] have?	13.13		X	
	111	Please tell us what was positive about your in experience with the in-person appointment(s) [baby number] had, and what could have been done better?	13.14		X	
	112	Please tell us what was positive about your in experience with the video appointment(s) [baby number] had, and what could have been done better?	13.15		X	
	113	Please tell us what was positive about your in experience with the telephone appointment(s) [baby number] had, and what could have been done better?	13.16		X	
	114	After the pandemic, how would you prefer to be seen for care of your child?	13.17		X	
	115	During the first year of life, have you needed to take [baby number] to Accident & Emergency (A&E), a walk-in centre, or an urgent care centre?	13.19		X	
	116	Why did you choose to take your baby for care at A&E, a walk-in centre, or an urgent care centre-?	13.2		X	
	117	Please explain your other reason	13.21		X	

Section	Row	Question	Baseline Question ID	Baseline notes	Follow-up Question ID	Follow-up notes
	118	How many other children (older or younger) are there who live at home with you and your baby?	13.22		X	
	119	The next questions are about your thoughts and feelings about your baby. Please tick one box only in answer to each question. There are 19 questions that should take about 10 minutes.	13.23		X	
	120	When I am caring for the baby, I get feelings of annoyance or irritation	13.24	MPAS 1	X	
	121	When I am caring for the baby I get feelings that the child is deliberately being difficult or trying to upset me	13.25	MPAS 2	X	
	122	Over the last two weeks I would describe my feelings for the baby as	13.26	MPAS 3	X	
	123	Regarding my overall level of interaction with the baby I	13.27	MPAS 4	X	
	124	When I interact with the baby I feel	13.28	MPAS 5	X	
	125	When I am with the baby I feel tense and anxious	13.29	MPAS 6	X	
	126	When I am with the baby and other people are present, I feel proud of the baby	13.3	MPAS 7	X	
	127	I try to involve myself as much as I possibly can PLAYING with the baby	13.31	MPAS 8	X	
	128	When I have to leave the baby	13.32	MPAS 9	X	
	129	When I am with the baby	13.33	MPAS 10	X	
	130	When I am not with the baby, I find myself thinking about the baby	13.34	MPAS 11	X	
	131	When I am with the baby	13.35	MPAS 12	X	
	132	When I have been away from the baby for a while and I am about to be with him/her again, I usually feel	13.36	MPAS 13	X	
	133	I now think of the baby as	13.37	MPAS 14	X	
	134	Regarding the things that we have had to give up because of the baby	13.38	MPAS 15	X	
	135	Over the past three months, I have felt that I do not have enough time for myself or to pursue my own interests	13.39	MPAS 16	X	

Section	Row	Question	Baseline Question ID	Baseline notes	Follow-up Question ID	Follow-up notes
	136	Taking care of this baby is a heavy burden of responsibility. I believe this is	13.4	MPAS 17	X	
	137	I trust my own judgement in deciding what the baby needs	13.41	MPAS 18	X	
	138	Usually when I am with the baby	13.42	MPAS 19	X	
	139	Do you think that your baby's development has been affected by lockdown?	13.43		X	
**Free text at end**	140	Is there anything else that you would like to share with us related to planning pregnancy, pregnancy, and the time after birth since February 2020 when the COVID-19 pandemic began in the UK?	10.2<br>12.2<br>13.44		Add	
	141	Interview study information	X		11.1	

GAD2 (General Anxiety Questionnaire 2-item), BP (general practitioner), MPAS (Maternal Postnatal Attachment Scale), PHQ2 (Patient Health Questionnaire-2)
